# Primitive Neural Stem Cells in the Adult Mammalian Brain Give Rise to GFAP-Expressing Neural Stem Cells

**DOI:** 10.1016/j.stemcr.2014.04.008

**Published:** 2014-05-22

**Authors:** Nadia Sachewsky, Rachel Leeder, Wenjun Xu, Keeley L. Rose, Fenggang Yu, Derek van der Kooy, Cindi M. Morshead

**Affiliations:** 1 The Donnelly Centre, University of Toronto, 160 College Street, Toronto, ON M5S 3E1, Canada; 2 Department of Surgery, University of Toronto, 160 College Street, Toronto, ON M5S 3E1, Canada; 3 Department of Molecular Genetics, University of Toronto, 160 College Street, Toronto, ON M5S 3E1, Canada; 4 Institute of Biomaterials and Biomedical Engineering, University of Toronto, 160 College Street, Toronto, ON M5S 3E1, Canada

## Abstract

Adult forebrain definitive neural stem cells (NSCs) comprise a subpopulation of GFAP-expressing subependymal cells that arise from embryonic fibroblast growth factor (FGF)-dependent NSCs that are first isolated from the developing brain at E8.5. Embryonic FGF-dependent NSCs are derived from leukemia inhibitory factor (LIF)-responsive, *Oct4*-expressing primitive NSCs (pNSCs) that are first isolated at E5.5. We report the presence of a rare population of pNCSs in the periventricular region of the adult forebrain. Adult-derived pNSCs (AdpNSCs) are GFAP^−^, LIF-responsive stem cells that display pNSC properties, including *Oct4* expression and the ability to integrate into the inner cell mass of blastocysts. AdpNSCs generate self-renewing, multipotent colonies that give rise to definitive GFAP^+^ NSCs in vitro and repopulate the subependyma after the ablation of GFAP^+^ NSCs in vivo. These data support the hypothesis that a rare population of pNSCs is present in the adult brain and is upstream of the GFAP^+^ NSCs.

## Highlights

• Rare, multipotent, self-renewing, *Oct4*^+^ AdpNSCs in the adult brain
• AdpNSCs lie upstream of definitive, GFAP-expressing adult NSCs
• AdpNSCs repopulate the SE after ablation of GFAP-expressing NSCs
• The AdpNSC pool is activated and expands after injury or LIF infusion in vivo

## Teaser

The identification of primitive NSCs that persist in the adult brain redefines the adult NSC lineage. Morshead and colleagues show that these adult-derived primitive NSCs, similar to primitive NSCs found in the developing embryo, express the pluripotency marker Oct4 in vitro and in vivo and give rise to GFAP^+^ definitive NSCs.

## Introduction

Neural stem cells (NSCs) in the adult brain reside in the periventricular region, where they generate progeny that migrate along the rostral migratory stream (RMS) to the olfactory bulb (OB) and become interneurons (Chojnacki et al., 2009; Garcia et al., 2004; Mirzadeh et al., 2008; Morshead et al., 1994). Numerous studies suggest that adult forebrain NSCs comprise a subpopulation of GFAP-expressing (GFAP^+^) cells within the subependyma (SE) lining the lateral ventricles (termed type B cells) (Capela and Temple, 2002; Chiasson et al., 1999; Doetsch et al., 1999a; Morshead et al., 2003). The GFAP^+^ NSCs generate clonally derived, multipotent, self-renewing colonies (termed neurospheres) in the presence of growth factors (epidermal growth factor [EGF] and fibroblast growth factor 2 [FGF2]) in vitro (Doetsch et al., 1999a; Imura et al., 2003; Morshead et al., 2003). The GFAP^+^ adult NSCs are derived from embryonic definitive NSCs and the two types of cells share similar properties, including FGF2 and EGF responsiveness, self-renewal, and multipotentiality (Hitoshi et al., 2004; Tropepe et al., 2001). GFAP expression in definitive NSCs occurs during development after embryonic day 16.5 (E16.5) (Imura et al., 2003) and continues into adulthood. Leukemia inhibitory factor (LIF)-dependent primitive NSCs (pNSCs) are present at E5.5 and give rise to FGF2-responsive NSCs beginning at E8.5, which then go on to generate GFAP^+^, neurosphere-forming, definitive NSCs in the adult brain.

We have found a rare population of pNSCs in the adult mammalian brain (termed AdpNSCs). We isolated cells from the adult periventricular region that generate clonally derived, self-renewing, and multipotent colonies in vitro in the presence of LIF. These LIF colonies can be passaged to give rise to GFAP^+^, neurosphere-forming cells in the presence of EGF and FGF2. Most interestingly, the LIF colonies expressed *Oct4* in vitro and contributed to the inner cell mass (ICM) of developing blastocysts after morula aggregation, which are characteristics attributed to pNSCs derived from embryonic stem cells (ESCs) (Hitoshi et al., 2004; Tropepe et al., 2001). We observed *Oct4* expression in periventricular tissue by quantitative PCR (qPCR) of primary cells and in whole-mount sections from adult brains. Further, we asked whether these AdpNSCs could generate GFAP^+^, neurosphere-forming NSCs in vivo. We took advantage of a transgenic mouse that expresses herpes simplex virus thymidine kinase under control of the GFAP promoter (termed GFAP-TK mice), which enabled the selective ablation of proliferating GFAP^+^ cells in vitro and in vivo (Bush et al., 1998, 1999; Imura et al., 2003; Morshead et al., 2003) after exposure to the antiviral agent ganciclovir (GCV). We used multiple ablation paradigms and found that after an initial and complete loss, GFAP^+^ NSCs invariably recovered over time, thereby confirming the presence of a GFAP^−^ cell upstream of the adult NSC in the lineage. Additionally, adult mice that are effectively AdpNSC null and do not generate LIF colonies are unable to repopulate the GFAP^+^ NSC population after ablation. Hence, these findings demonstrate the presence of a rare population of *Oct4*^+^ pNSCs in the adult forebrain whose progeny include GFAP^+^ type B cells that are indeed neurogenic in vivo (Doetsch et al., 1999a) and form neurospheres in vitro (Morshead et al., 2003).

## Results

### Multipotent and Self-Renewing LIF-Responsive Colony-Forming Cells Are Present in the Periventricular Region

**Figure 1:**
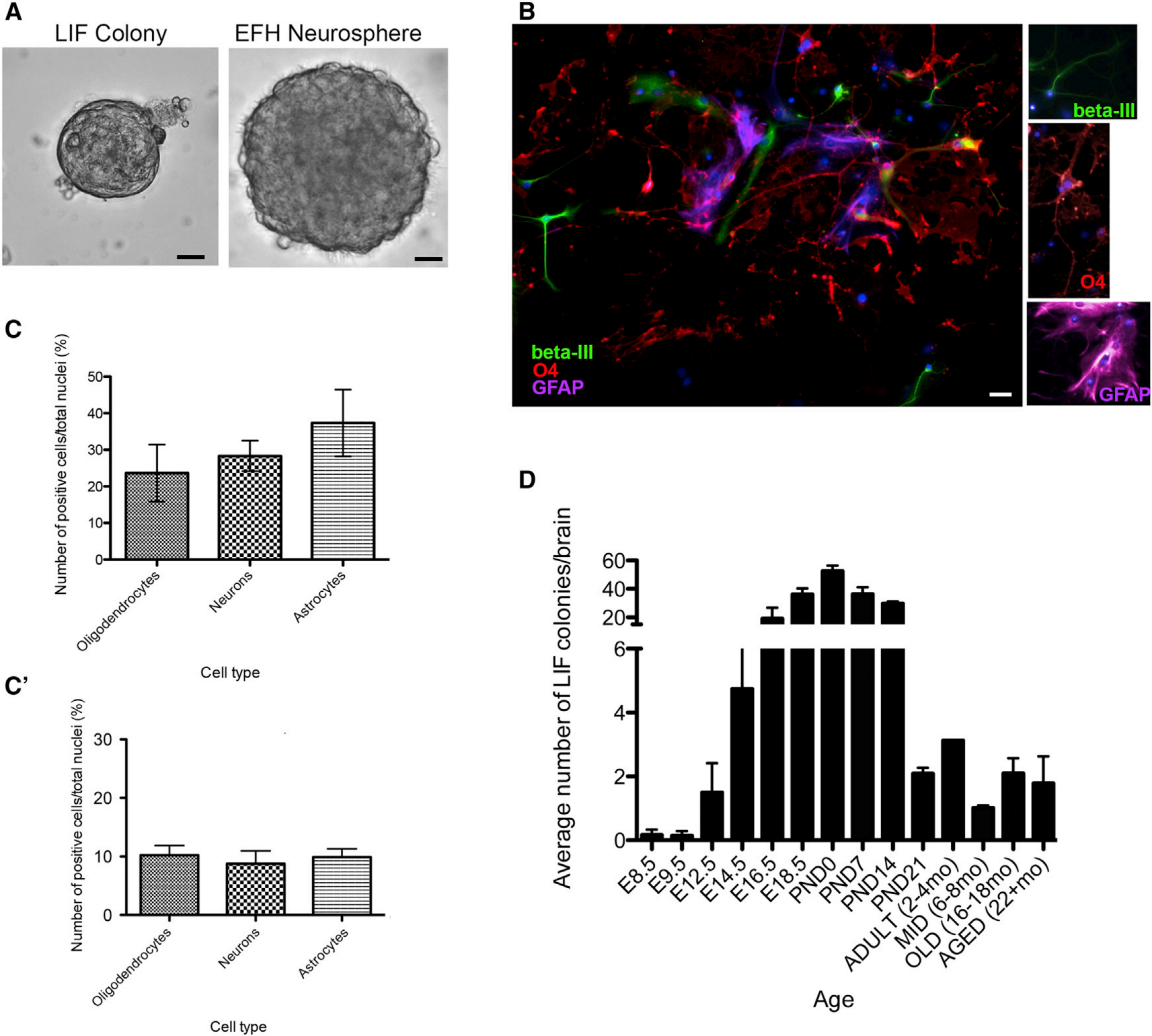
LIF-Responsive Colonies Are Derived from the Adult Periventricular Region (A) Spherical, free-floating colonies (50–150 μm in diameter) were observed in LIF and compared with neurospheres grown in EFH (>100 μm diameter). (B) LIF-responsive colonies gave rise to neurons (BIII tubulin^+^, green), astrocytes (GFAP^+^, purple), and oligodendrocytes (O4^+^, red). (C) Differentiation profile of LIF colonies reveals that oligodendrocytes, neurons, and astrocytes are produced at equal frequency upon differentiation (similar to what is seen in ESC-derived pNSC colonies (C’) (n ≥ 3 colonies per group, >400 cells per colony were counted). (D) Free-floating LIF colonies could be isolated at all time points throughout development and into old age (n > 4 per time point). Data are shown as mean ± SEM. Scale bars represent 40 μm (A) and 50 μm (B). See also Figure S1.

Studies of mouse neural development have indicated the presence of a LIF-dependent pNSC that gives rise to FGF2-dependent NSCs in the early embryo (Akamatsu et al., 2009; Bauer and Patterson, 2006; Doetsch et al., 1999b); thus, we investigated the potential continued presence of a LIF-responsive cell in the adult forebrain. We observed LIF receptor-positive (LIFR^+^) cells in the ependyma and SE of the adult brain, similar to previous observations (Bauer and Patterson, 2006; Figure S1A available online). We prepared neurosphere cultures at clonal densities (Coles-Takabe et al., 2008) from adult mice in the presence of LIF alone, which is identical to conditions used to isolate pNSCs from the early embryonic brain (Hitoshi et al., 2004). We observed a rare population of free-floating spherical colonies of cells with well-defined borders in LIF-only conditions (1.5 ± 0.2 per 40,000 cells) that were >50 μm in size but typically <150 μm (Figure 1A). The low incidence of adult LIF-only colony formation is consistent with the rare frequency of pNSCs during early development (Hitoshi et al., 2004). Individual adult LIF colonies could be passaged into LIF-only for more than four passages and differentiated into neurons, astrocytes, and oligodendrocytes, thereby displaying the properties of self-renewal and multipotentiality (Figure 1B). Adult LIF colonies differentiated into all three neural phenotypes with equal frequencies (Figure 1C), similar to in vitro ESC-derived pNSCs (Figure 1C’). This is significantly different from adult EFH (EGF, FGF2, and heparin dependent) neurospheres, in which astrocytes comprise the vast majority of differentiated progeny (Hunt et al., 2010). Hence, the differentiation profiles support the conclusion that pNSCs, regardless of the age of derivation, are more similar to each other than to the definitive NSCs. Notably, dissections of the striatum and cortex did not result in LIF colony formation. Taken together, these data reveal that the adult brain periventricular region contains a stem cell population that is distinct from the definitive GFAP^+^, EFH-responsive NSC in terms of its differentiation potential and LIF dependence.

To determine whether this LIFR^+^ stem cell population was distinct from the adult GFAP^+^ NSCs, we employed a transgenic mouse that expresses thymidine kinase from the GFAP promoter (GFAP-TK), thereby permitting the specific ablation of dividing GFAP^+^ cells in the presence of GCV in vivo and in vitro (Morshead et al., 2003). GCV is taken up by all cells and phosphorylated by the transgene, leading to the accumulation of toxic metabolites and cell death when the GFAP-expressing cells undergo mitosis. When primary cultures from GFAP-TK mice were grown in standard neurosphere conditions (EFH), no neurospheres formed in the presence of GCV (Figure 4A), indicating that neurospheres were derived from GFAP^+^ cells as previously observed (Morshead et al., 2003). In sharp contrast, primary LIF colonies from GFAP-TK mice formed even in the presence of GCV in vitro and at the same frequency as LIF colonies from nontransgenic (NT) controls (1.1 ± 0.5 versus 1.3 ± 0.6 colonies per 40,000 cells for GFAP-TK and NT, respectively), indicating that the LIF-responsive colonies are derived from a GFAP^−^ cell. Most interestingly, we found that individual colonies grown in LIF alone, from GFAP-TK and NT mice, could be passaged into EFH, indicating that LIF colonies (derived from GFAP^−^ cells) were able to generate GFAP^+^ neurosphere-forming cells during colony formation. Notably, LIF colonies from GFAP-TK mice could not be passaged into EFH and GCV, indicating that the secondary neurospheres that formed in EFH were derived from GFAP^+^ cells. Together, these data reveal that a LIFR^+^, GFAP^−^, colony-forming stem cell is present in the adult brain and is able to generate GFAP^+^ type B cells that form EFH neurospheres in vitro.

We examined the lineage relationship between the LIF-responsive free-floating colonies and EFH neurospheres. Neurospheres were derived from wild-type animals under standard neurosphere conditions (free floating in serum-free media containing EFH or EGF-only). Individual clonally derived neurospheres were collected, dissociated, and replated to assess secondary neurosphere formation. Invariably, individual primary EFH neurospheres (derived from the GFAP^+^ adult NSCs) passaged into EFH generated secondary neurospheres that were subsequently propagated through more than five passages (n = 18/18 neurospheres). In contrast, individual primary EFH neurospheres never passaged into LIF-only conditions (n = 0/18). These findings indicate that the GFAP^+^ adult NSC does not give rise to AdpNSCs.

We asked whether EGF-only neurospheres could be passaged into LIF conditions to form secondary neurospheres and, again, we never observed secondary neurospheres from individual EGF-only neurospheres (n = 0/18 individual neurospheres). Most importantly, and in contrast to previous reports that EGF neurospheres are derived from GFAP^−^ transit-amplifying cells (type C cells) (Doetsch et al., 2002), our EGF-only primary neurospheres from GFAP-TK animals never passaged into EGF and GCV (n = 0/12), indicating that EGF-only neurospheres are derived from a GFAP-expressing adult NSC. Taken together, these results indicate that our adult-derived LIF colonies are derived from GFAP^−^ stem cells that can give rise to GFAP^+^ adult NSCs, and further, that the reverse relationship is not true: GFAP^+^ adult NSCs do not give rise to AdpNSCs in vitro.

We next asked whether this adult population of LIF-responsive NSCs was seen throughout embryonic development and into adulthood. Previous work demonstrated that embryonic pNSCs declined by E8.5 to barely detectable levels (Hitoshi et al., 2004); however, they did not extend their findings to later times in embryogenesis. Here, we isolated primary periventricular tissue at various developmental stages, starting at E8.5, and cultured the cells in LIF conditions. We found that LIF-responsive free-floating colonies could be isolated at E8.5 at rare frequencies (Figure 1D), consistent with what was shown by Hitoshi et al. (2004), and further, that the population expanded in number during late embryogenesis and the early postnatal period, followed by a decrease into adulthood that was maintained into old age (>22 months; Figure 1E). Hence, LIF-responsive pNSCs can be isolated from both the developing and the adult brain.

### LIF Colonies Express *Oct4* In Vitro and In Vivo and Integrate into the ICM of Blastocysts

**Figure 2:**
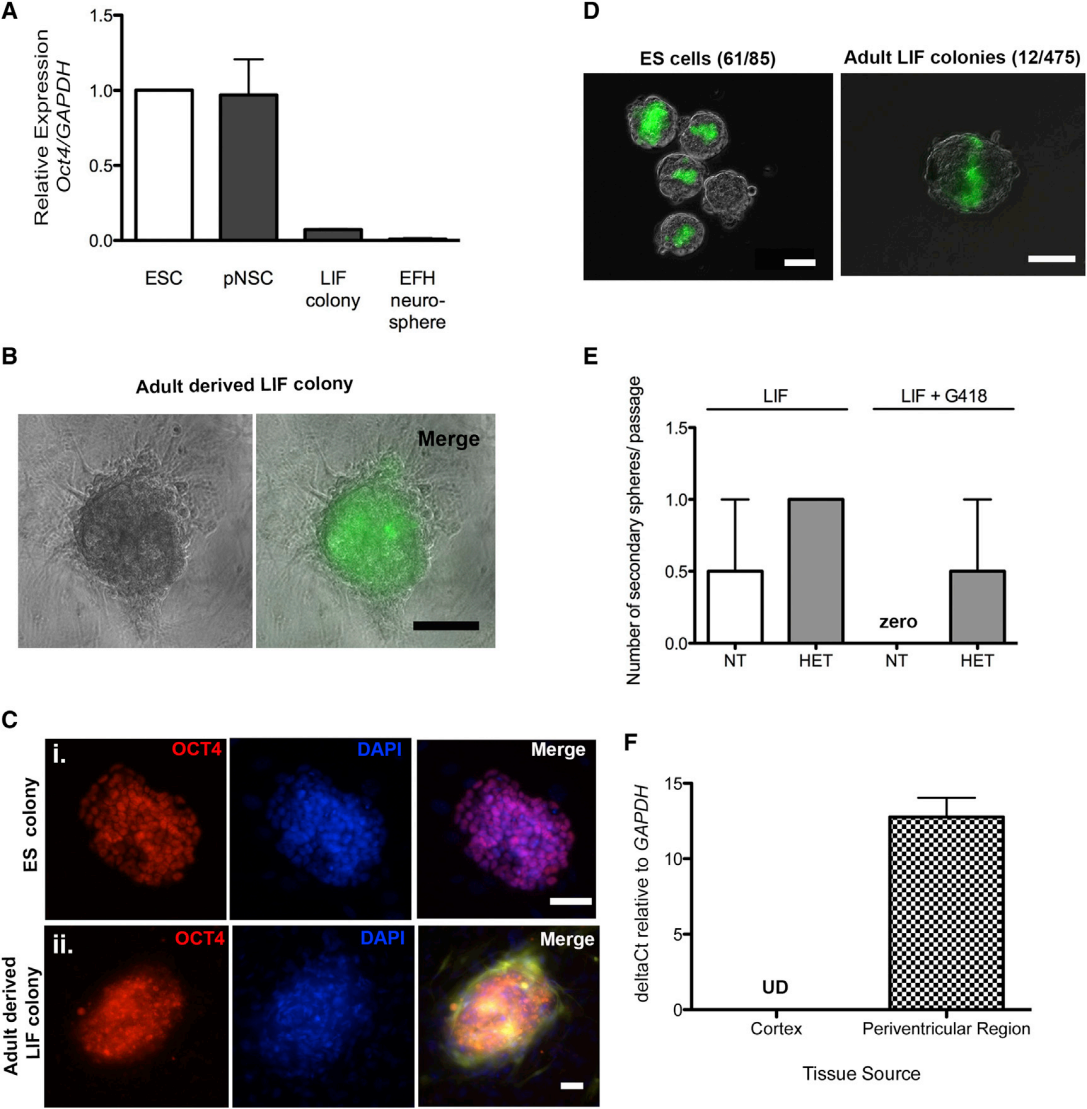
LIF Colonies Express *Oct4* and Integrate into the ICM of Blastocysts (A) qPCR *Oct4* expression in ESCs, pNSC, adult LIF colonies grown in ESC conditions, and adult neurospheres grown in EFH (n = 3 independent samples/group, in triplicate). (B) LIF colonies from *Oct4*-GFP mice grown in ESC conditions express *Oct4* (green). (C) Control ESC colonies (i) and adult LIF colonies (ii) from YFP-expressing mice grown in ESC conditions express OCT4 (red). (D) ESCs and adult LIF colony cells (green) integrate into the ICM of blastocysts. (E) LIF colonies derived from *Oct4*-neo transgenic mice, but not from control animals, form secondary colonies in G418 (n = 12 passaged colonies/group). (F) qPCR *Oct4* expression from the periventricular region of adult brain compared with control (n = 4/group). Data are shown as mean ± SEM. Scale bars represent 100 μm (B), 50 μm (Ci), 25 μm (Cii), and 50 μm (D). See also Movie S1.

We asked whether the LIFR^+^ colony-forming cells in the adult brain had properties similar to pNSCs derived from the embryonic brain. pNSCs derived from E5.5–8.5 embryos or from ESCs display properties of pluripotency, including the expression of *Oct4* (Akamatsu et al., 2009), and pNSCs derived from ESCs have the capacity to integrate into the ICM of blastocyst chimeras (Tropepe et al., 2001). We grew adult-derived LIF colonies in ESC conditions (on mouse embryonic fibroblast [MEF] feeder cells) and generated colonies morphologically similar to ESC colonies. The adult LIF colonies expressed *Oct4*, as revealed by qPCR, immunohistochemistry, and GFP expression, when cells were derived from *Oct4*-GFP transgenic mice (Figures 2A–2C). We made morula aggregates using transgenic yellow fluorescent protein (YFP)-expressing cells from adult mice grown as (1) LIF colonies on MEFs, (2) neurospheres in standard EFH conditions (negative control), and (3) YFP-expressing ESCs (positive control). Consistent with previous studies, ESCs integrated at a high frequency (71.7%), whereas adult EFH neurosphere-derived cells did not integrate into the developing blastocysts in vitro (Karpowicz et al., 2007). Most strikingly, adult LIF-colony-derived cells also integrated into the ICM, albeit at a much lower frequency (2.5%; Figure 2D). The rare frequency of integration may be the result of the heterogeneous population within a LIF colony consisting of both pluripotent AdpNSCs and definitive GFAP^+^ NSCs. This likely leads to an underestimation of the true number of AdpNSCs that can integrate into chimeric blastocysts. To date, we have not observed any AdpNSC chimeras that survive until E9.5 after they are transferred back to pseudopregnant mice, which perhaps is consistent with the lower level of *Oct4* expression in AdpNSC colonies compared with ESCs and embryonic pNSCs (Figure 2A).

We grew adult LIF colonies in LIF alone (no feeders) from *Oct4*-neo transgenic mice, which harbor a neomycin resistance cassette knocked into the *Oct4* locus, thereby conferring neomycin resistance in *Oct4*-expressing cells. After passaging individual *Oct4*-neo primary LIF colonies, we observed the formation of secondary LIF colonies in the presence of neomycin, revealing that LIF colonies were derived from *Oct4*-expressing cells (Figure 2E). As predicted, primary neurospheres derived in EFH could never be passaged in the presence of neomycin. This complete depletion of EFH neurospheres, but unchanged numbers of LIF colonies, indicates appropriate antibiotic selection of *Oct4*^+^ cells in the presence of G418. If variable expression of the transgene was occurring, we also would expect there to be some *Oct4* cells that did not possess the antibiotic resistance gene and therefore would die in the presence of the antibiotic. Notably, no such death was seen. Taken together, these findings reveal that adult-derived LIF colonies display properties similar to those of pNSCs from the developing embryo.

**Figure 3:**
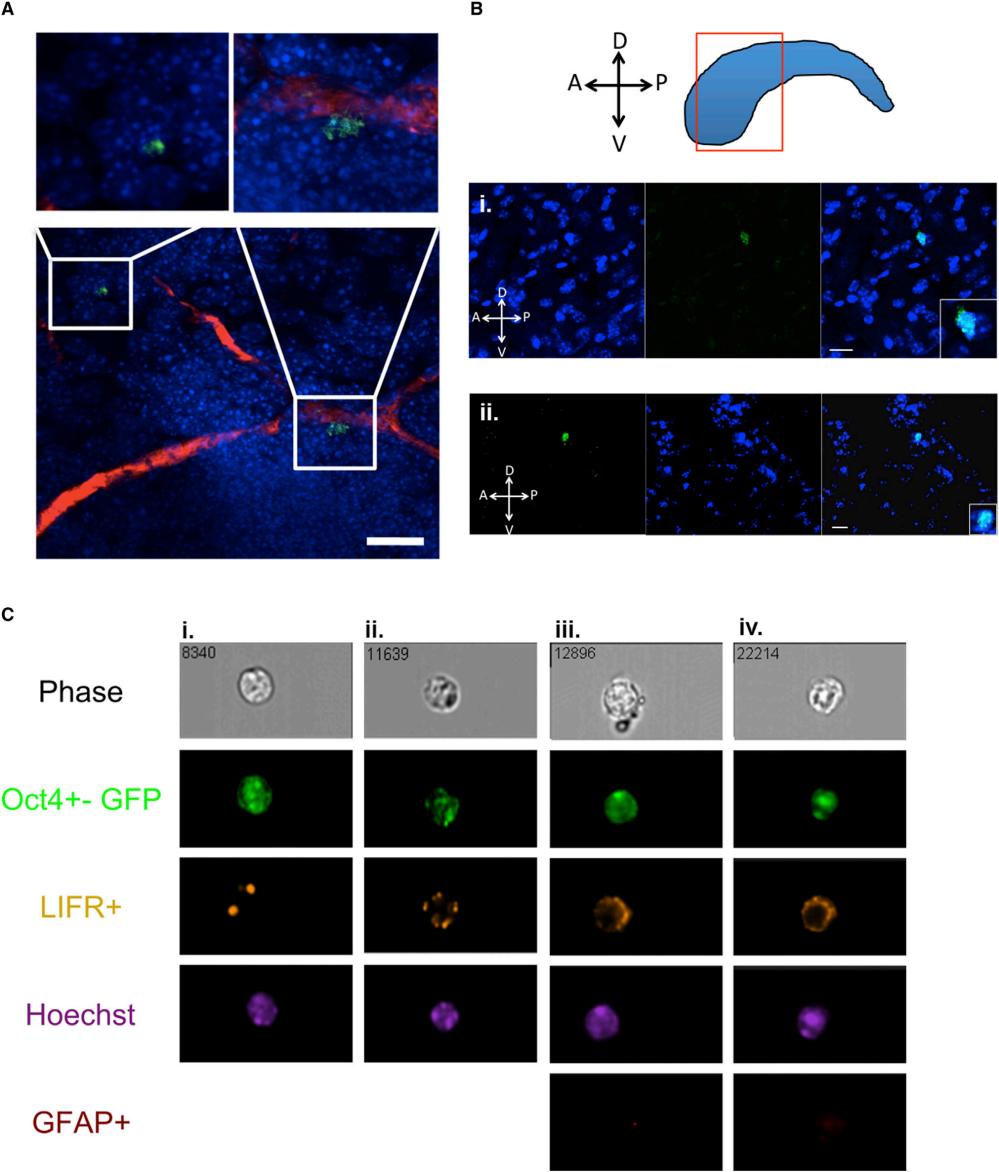
*Oct4*-Expressing Cells Are Present In Vivo (A) Whole-mount sections derived from *Oct4*-GFP mice reveal *Oct4*^+^ (green) periventricular cells (DAPI, blue punctate nuclei) optimized to detect GFP expression in high-magnification images in the adult brain (blood vessels, red). (B) Whole-mount sections of the periventricular region of *Oct4*-GFP adult mice reveal *Oct4*^+^ (green) cells labeled with Hoechst (blue). Insets show increased magnification of the *Oct4*^+^ cells. Orientation markers provided within images. Scale bar, 20 μm. (C) Image-stream images showing (i and ii) *Oct4*^+^ LIFR^+^ live cells and (iii and iv) *Oct4*^+^ LIFR^+^ GFAP^−^ fixed cells from *Oct4*-GFP mice. See also Figure S2.

To ensure that the *Oct4* expression observed in vitro was not an artifact of culturing periventricular cells, we looked for *Oct4* expression in vivo. Given that AdpNSCs represent an exceedingly rare population of cells and their *Oct4* expression is extremely low, we performed qPCR on primary dissected periventricular tissue. We observed significant *Oct4* mRNA expression in the periventricular tissue, in contrast to a complete lack of expression in cortical tissue from the adult brain (Figure 2F). We also looked for the presence of GFP-expressing cells in the periventricular region from *Oct4*-GFP-expressing mice and observed rare GFP cells in whole-mount sections of the lateral ventricle periventricular region (Figures 3A and 3B; Movie S1). We identified rare GFP-expressing cells in the periventricular region (Figure S2B) that colocalized with LIFR, but not with GFAP or β-catenin, consistent with our in vitro findings. Furthermore, we performed a fluorescence-activated cell sorting (FACS) analysis of primary dissections of the periventricular region from adult *Oct4*-GFP mice to estimate the number of *Oct4*^+^ AdpNSCs. The FACS analysis revealed that 0.08% of the sorted cells were *Oct4*^+^, suggesting that approximately 80 cells in the periventricular region are AdpNSCs (100,000 cells are obtained per brain dissection). This estimate is higher than might be expected from the neurosphere assay and suggests that we may not have optimized our AdpNSC culture conditions and/or that the increased sensitivity of FACS to levels of GFP expression led to the isolation of GFP^+^ cells that were not visualized in whole-mount sections. To supplement our FACS findings, we utilized an imager coupled with flow cytometry to visually identify single cells. Live-cell staining identified GFP^+^ and LIFR^+^ populations from *Oct4*-GFP mice (Figure 3Ci and ii). We used fixed cells from *Oct4*-GFP mice and showed that all GFP^+^ and LIFR^+^ cells were always GFAP^−^ (Figure 3Ciii and iv), consistent with our whole-mount data (Figure S2B). Most importantly, we never observed a GFP-expressing cell from control tissue (the periventricular region of CD1 control mice or the cortex of *Oct4*-GFP mice). Together, these findings demonstrate that *Oct4*-expressing cells are present in the periventricular region of the adult brain in vivo.

To further characterize the AdpNSCs, we examined the gene expression of AdpNSCs using qPCR on LIF colonies, EFH neurospheres, and ESCs. We looked at additional markers of pluripotency, including *Nanog*, *SOX2*, *Klf4*, *TERT*, and *c-myc* (Figure S2A). We observed that LIF colonies expressed detectable levels of *Nanog*, which was undetectable in EFH neurospheres. Furthermore, LIF colonies expressed mRNA levels of *Klf4* and *c-myc* equivalent to those observed in ESCs (0.88- and 1.7-fold relative to ESCs, respectively). *SOX2* and *TERT* were also identified in LIF-responsive colonies (0.54- and 1.7-fold expression relative to ESCs, respectively). Examination of proneural markers, including *SOX1*, *Notch*, *Nestin*, and *CD133* (Figure S2A), revealed that LIF colonies expressed significantly lower mRNA levels of the neural markers *SOX1*, *Notch*, and *Nestin* compared with EFH neurospheres. *CD133* was similar in LIF colonies and EFH neurospheres (Figure S2A). Thus, we report that adult LIF colonies have higher expression of pluripotency markers and lower levels of definitive NSC markers, making them more ESC-like. Taken together, the immunohistochemistry, whole-mount, and qPCR data reveal that AdpNSCs are LIFR^+^/*Oct4*^+^/GFAP^−^/*SOX2*^+^*/Nestin*^+^*/CD133*^−^.

### AdpNSCs Repopulate GFAP-Expressing NSCs after Ablation with GCV In Vivo

**Figure 4:**
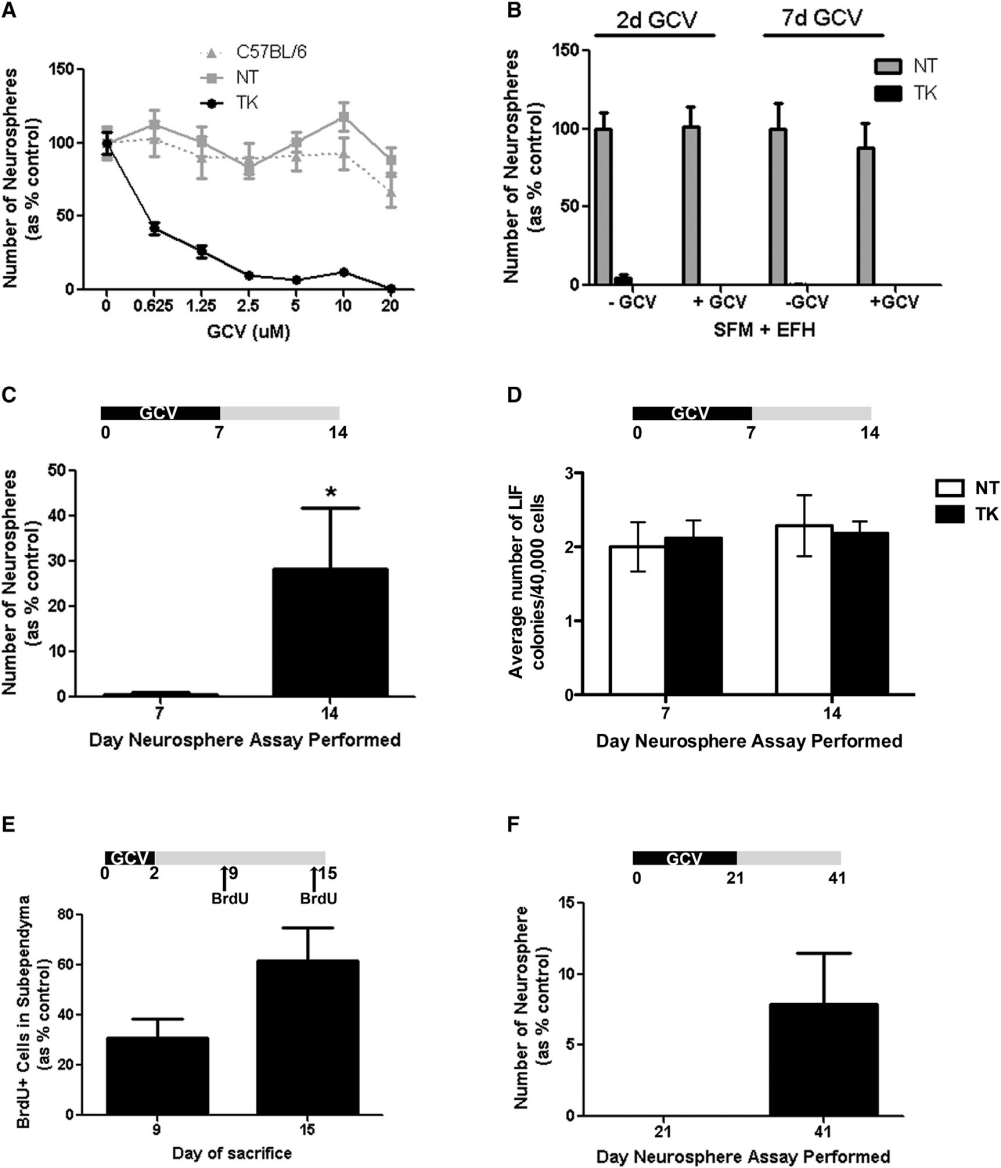
GFAP-TK Model Specifically Ablates Dividing GFAP^+^ Cells In Vitro and In Vivo (A) GCV dose response curve in vitro (n = 2 independent experiments). (B) Two- or 7-day GCV infusion in vivo followed by immediate sacrifice with cells plated in EFH in the absence (−) or presence (+) of GCV in vitro (n ≥ 5 mice/group). (C and D) Seven-day GCV infusion (n ≥ 3 mice/group) followed by plating in (C) EFH to assay GFAP-expressing adult NSCs or (D) LIF-only to assay for AdpNSCs. (E) BrdU^+^ cells in the SE after 2-day GCV infusion (n = 3 mice/group). (F) EFH neurosphere assay after 21-day GCV infusion (n = 3 mice/group). Data are shown as mean ± SEM; ^∗^p ≤ 0.05. See also Figure S3.

Based on the NSC lineage from embryonic development into adulthood, we asked whether AdpNSCs give rise to GFAP^+^ neurosphere-forming NSCs in the adult brain. To address this question, we performed in vivo experiments to examine the potential for AdpNSCs to contribute to SE repopulation using the GFAP-TK mouse. Previous studies using GFAP-TK transgenic mice led us to predict that intraventricular infusion of GCV would effectively create a GFAP^+^ NSC-depleted mouse, as cells would be killed while proliferating (Bush et al., 1998, 1999; Imura et al., 2003; Morshead et al., 2003). In vitro, we observed a complete loss of clonal EFH neurosphere formation from the SE of GFAP-TK mice in the presence of 20 μM GCV, with no effect of GCV on the number (Figure 4A) or size (Figure S3A) of NT littermate control EFH neurospheres. In vivo, intraventricular GCV infusions into GFAP-TK and NT mice for 7 days, followed by immediate sacrifice, resulted in GFAP^+^ NSCs depletion, with a ≥99.5% ± 0.5% loss in clonal EFH neurosphere formation from GFAP-TK versus NT mice (Figures 4B and 4C), similar to previous findings (Garcia et al., 2004; Morshead et al., 2003). However, when the neurosphere assay was performed on GFAP-TK mice that survived for various times after GCV infusion, the numbers of EFH neurospheres returned over time, reaching 30% of control levels by day 14 (7 days after GCV treatment; Figure 4C) despite an initial 99.5% depletion in EFH neurosphere numbers at the time of sacrifice (one in six animals examined had one EFH neurosphere). A similar return of EFH neurospheres was seen after 2 days of GCV infusion (Figure S3C). Significantly, in vitro exposure to GCV completely and invariably eliminated EFH neurosphere formation from GFAP-TK mice at all times examined (at immediate sacrifice and during EFH neurosphere recovery postinfusion), indicating that the returning EFH neurospheres were derived from GFAP^+^ cells. Furthermore, neurospheres never grew in EGF alone immediately after the GCV infusions or when cultured in EGF and GCV at later survival times, indicating that GFAP^−^, EGF-responsive progenitor cells (Doetsch et al., 2002) were not responsible for the return of neurospheres over time (Figure S3B). Interestingly, after GCV infusion for 2 or 7 days in vivo, the number of AdpNSC LIF colonies that formed in vitro was unchanged upon immediate sacrifice and at later survival times after GCV infusion (Figure 4D). These findings indicate that the LIF colonies are derived from a GFAP^−^ cell, as they are not depleted by GCV ablation. Further, the LIF population divides asymmetrically to repopulate the GFAP^+^ NSCs in vivo, since the number of LIF colonies does not expand after the ablation. Consistent with the return of EFH neurospheres in vitro, the number of proliferating cells (BrdU^+^) in vivo in GCV-infused GFAP-TK mice increased with longer survival times (Figure 4E).

We extended the length of GCV infusion in vivo to 21 days, which is 50% longer than the estimated 15-day cell-cycle time of slowly dividing adult NSCs (Morshead et al., 1998), to eliminate the possibility that a rare GFAP^+^ NSC escaped the GCV treatment and repopulated the SE. We observed a complete, 100% loss of GFAP^+^ NSC-derived, EFH neurosphere formation after 21 days of GCV treatment; however, EFH neurospheres still returned with longer survival times (Figure 4F). The invariable return of GFAP^+^ NSCs over time supported the hypothesis that an earlier cell in the lineage that was GFAP^−^ was able to repopulate the SE.

### In Vivo Ablation of GFAP^+^ Cells after AraC Activation Does Not Lead to a Permanent Loss of NSCs

**Figure 5:**
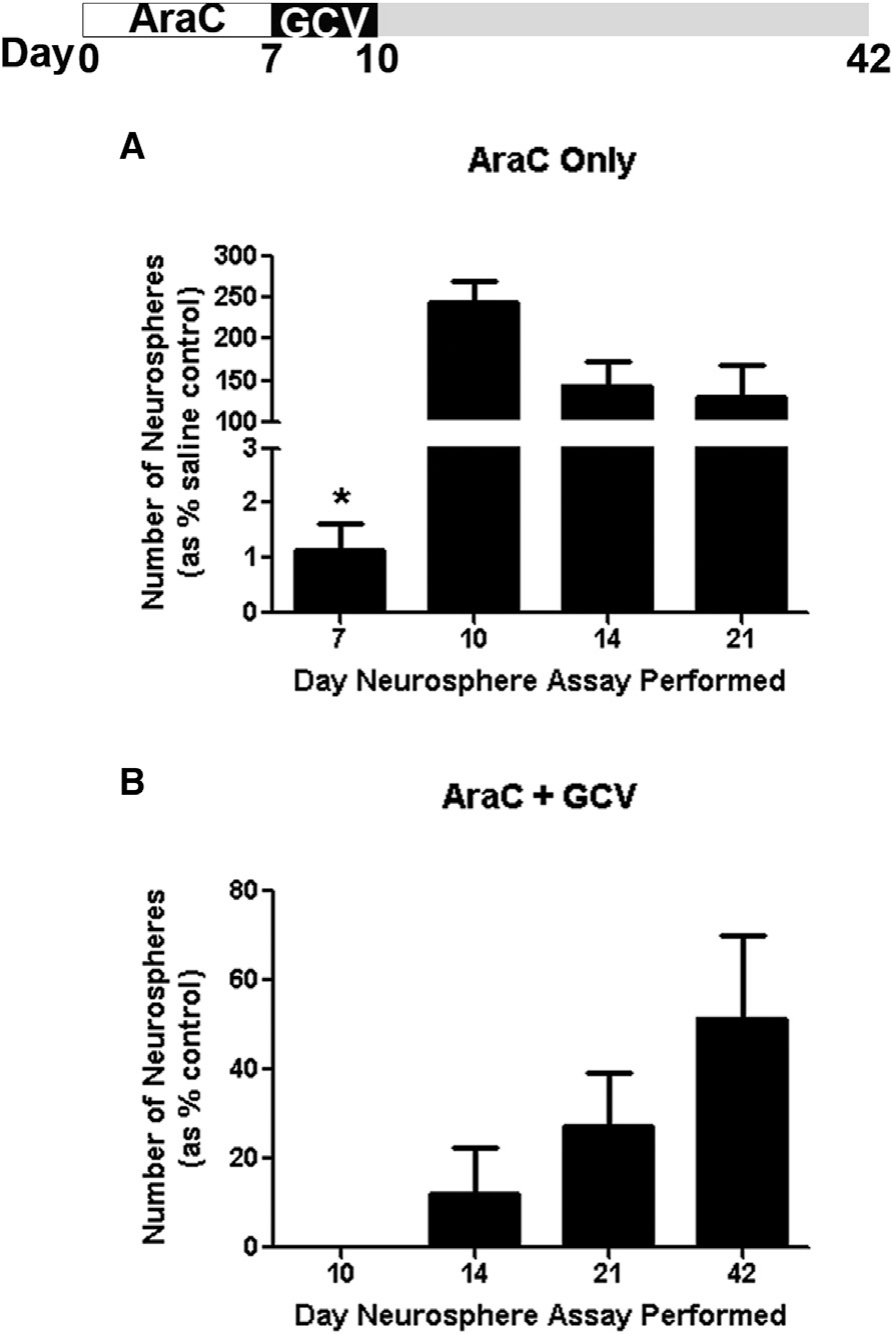
Infusion of AraC and GCV Leads to Complete but Temporary Loss of Neurospheres (A) AraC-only infusion for 7 days into TK mice resulted in a 99% ± 0.5% loss in neurospheres relative to saline-infused controls when sacrificed immediately after infusion (day 7). Neurospheres returned to control values by 3 days after AraC infusion (n = 3–6 mice/group/time point). (B) AraC and GCV infusion eliminated TK neurosphere formation immediately after the infusion (day 10), but returned with longer survival times (n = 3–5 mice/group). Controls are NT mice that received infusions. Data are shown as mean ± SEM; ^∗^p < 0.0001. See also Figure S4.

We performed more rigorous attempts to permanently deplete GFAP^+^ NSCs in vivo using a well-established paradigm to induce GFAP^+^ NSC proliferation prior to the administration of GCV in vivo. Previous work has shown that infusion of an antimitotic agent kills the rapidly proliferating NSC progeny in the SE and leads to the recruitment and division of GFAP^+^ NSCs, with repopulation of the SE within 8–10 days (Doetsch et al., 1999b; Morshead et al., 1994). Based on these findings, we infused cytosine β-D-arabinofuranoside (AraC) intraventricularly for 7 days, followed immediately by GCV infusion during the time when GFAP^+^ NSCs are proliferating to repopulate the SE (Doetsch et al., 1999b; Morshead et al., 1994). Similar to previous findings (Doetsch et al., 2002), the number of EFH neurospheres observed after AraC infusion alone was initially depleted but quickly returned to control values (Figure 5A). Indeed, the number of GFAP^+^ NSCs in AraC-treated animals was 1.6-fold greater than in saline-infused controls at 3 days postinfusion (day 10 sacrifice), indicating an initial overcompensation as the surviving GFAP^+^ NSCs underwent expansionary divisions to repopulate the SE. In contrast, GFAP-TK mice that received AraC and GCV treatment for 3 days revealed a 100% loss of neurosphere formation upon immediate sacrifice (day 10) and a slower return of GFAP^+^ NSCs. EFH neurosphere formation inevitably returned at longer survival times, but did not return to control levels at the longest time examined (51.4% ± 18.6% of controls by day 42; Figure 5B). Extending the GCV infusion to 7 days after AraC treatment revealed a similar initial 100% loss of EFH neurosphere formation followed by a return at longer survival times (21.5% ± 11.5% of NT controls by day 43). Most importantly, the addition of GCV in vitro resulted in a complete and invariable loss of EFH neurosphere formation from GFAP-TK mice at all survival times, indicating that the in vitro EFH neurospheres that returned over time were generated from GFAP^+^ cells. Thus, despite using multiple well-established kill paradigms to specifically ablate dividing GFAP^+^ cells in vivo, we were unable to permanently deplete clonal EFH neurosphere formation, indicating that GFAP^+^ cells recovered in vivo over time.

The loss of EFH neurosphere-forming cells, albeit temporary, should also result in an initial loss of proliferating NSC progeny. We examined the numbers of proliferating cells in vivo after the 7-day AraC and 7-day GCV infusion. As predicted, we observed an initial loss, followed by a complete recovery, of the numbers of proliferating cells by day 42 posttreatment in the SE, RMS, and OB (Figures S4 and S5A). Further, after the AraC and GCV infusion, we observed proliferating LIFR^+^ cells in the SE (Figures S1B–S1D). Hence, despite the in vivo depletions with AraC and GCV treatment, the numbers of GFAP^+^, neurosphere-forming cells and their progeny in vivo returned over time.

We asked whether the inability to achieve permanent depletion of EFH neurospheres in vitro, or proliferating cells in vivo, was due to GCV degradation in GFAP^+^ NSCs over time, thereby leading to neurosphere formation with longer survival times. We determined the length of time that GCV remains effective at killing proliferating GFAP^+^ cells once it is taken up by cells. Astrocyte monolayers from early postnatal cortices of GFAP-TK and NT mice demonstrated that GCV remains toxic to cells for at least 10 days after a single 16 hr exposure to the drug (Figures S5B and S5C), which exceeds the in vivo survival times postinfusion when we observed the return of neurospheres in vitro. Hence, a loss of GCV toxicity cannot account for the return of neurospheres over time. Thus, these data provide strong support for the presence of an upstream stem cell that is GFAP^−^ and capable of generating GFAP^+^ NSCs after ablation in vivo.

Finally, we studied the lineage relationship between AdpNSCs and GFAP^+^ adult NSCs using a transgenic Floxed *Oct4*-*Sox1*Cre mouse, which allows for specific ablation of the *Oct4* population in cells that express the neural gene *Sox1*. Floxed *Oct4*-*Sox1*Cre mice are devoid of AdpNSCs and never give rise to LIF colonies in vitro, whereas the littermate controls generate normal numbers of LIF colonies (2.8 ± 0.4 per 40,000 cells) and EFH neurospheres are not changed (25.3 ± 3.7 versus 27.5 ± 4.0 neurospheres per 5,000 cells from transgenic versus littermate controls, respectively). The Floxed *Oct4*-*Sox1*Cre mice have normal numbers of EFH neurospheres, likely because *Sox1* expression turns on after pNSCs and definitive NSCs are present in the developing brain (Wood and Episkopou, 1999), and hence the *Oct4* allele is excised after the neural lineage is established. Most interestingly, after ablation of the EFH neurospheres, the preliminary data revealed a lack of GFAP^+^ adult NSC repopulation (0 ± 0 EFH neurospheres versus 10.2 ± 0.1 EFH neurospheres per 5,000 cells from transgenic versus littermate controls, respectively). These findings indicate that AdpNSCs are necessary for repopulating the EFH-responsive, GFAP^+^ adult NSC in vivo after ablation in the adult brain, and support the hypothesis that GFAP^+^ adult NSCs are the progeny of the AdpNSCs.

### Transplanted LIF-Responsive Colonies, Devoid of GFAP-Expressing Adult NSCs, Contribute to Neurogenesis In Vivo

Based on our hypothesis that AdpNSCs generate GFAP^+^ adult NSCs, we predicted that AdpNSC progeny would contribute to neurogenesis in vivo. We performed transplantation experiments to test our hypothesis using populations of AdpNSCs that did not contain GFAP^+^ progeny prior to transplantation. GFAP-TK mice were crossed to a YFP reporter mouse to generate mice (YFP-GFAP-TK mice) that ubiquitously express YFP and permit the selective ablation of dividing GFAP^+^ cells in the presence of GCV. When LIF colonies from YFP-GFAP-TK mice were isolated in vitro, the total number of LIF colonies was the same in the presence or absence of GCV and did not differ from NT YFP controls grown in GCV. However, the YFP-GFAP-TK LIF colonies were smaller in size than control LIF colonies, likely due to ablation of the GFAP-expressing progeny within the colony (Figures S6A and S6B). As predicted, YFP-GFAP-TK LIF colonies grown in the presence of GCV never generated EFH neurospheres, whereas those grown in the absence of GCV, or LIF colonies grown in the presence of GCV from NT littermate controls, always gave rise to EFH (Figure S6C). Thus, the YFP-GFAP-TK-derived LIF colonies did not contain GFAP^+^ neurosphere-forming cells prior to transplantation. We generated single-cell suspensions from 2-week-old LIF and GCV colonies and transplanted 800 YFP^+^ cells into the anterior SE of wild-type CD1 mice. We examined the mice at 48 hr postinjection or 14 days postinjection. At early sacrifice, 4.6% of transplanted YFP^+^ cells had survived and were observed at the injection site (Figure 7A). At day 14 posttransplantation, we observed YFP^+^ cells migrating along the RMS (Figures 7Ci and ii and S6Di) and residing in the OB (Figure 7Ciii), as well as differentiating into a neuronal-like morphology (Figures S6Dii and iii). These data further support the hypothesis that AdpNSCs are able to contribute to neurogenesis in vivo.

### The AdpNSC Population Is Activated and Expanded after Stroke or LIF Infusion

**Figure 6:**
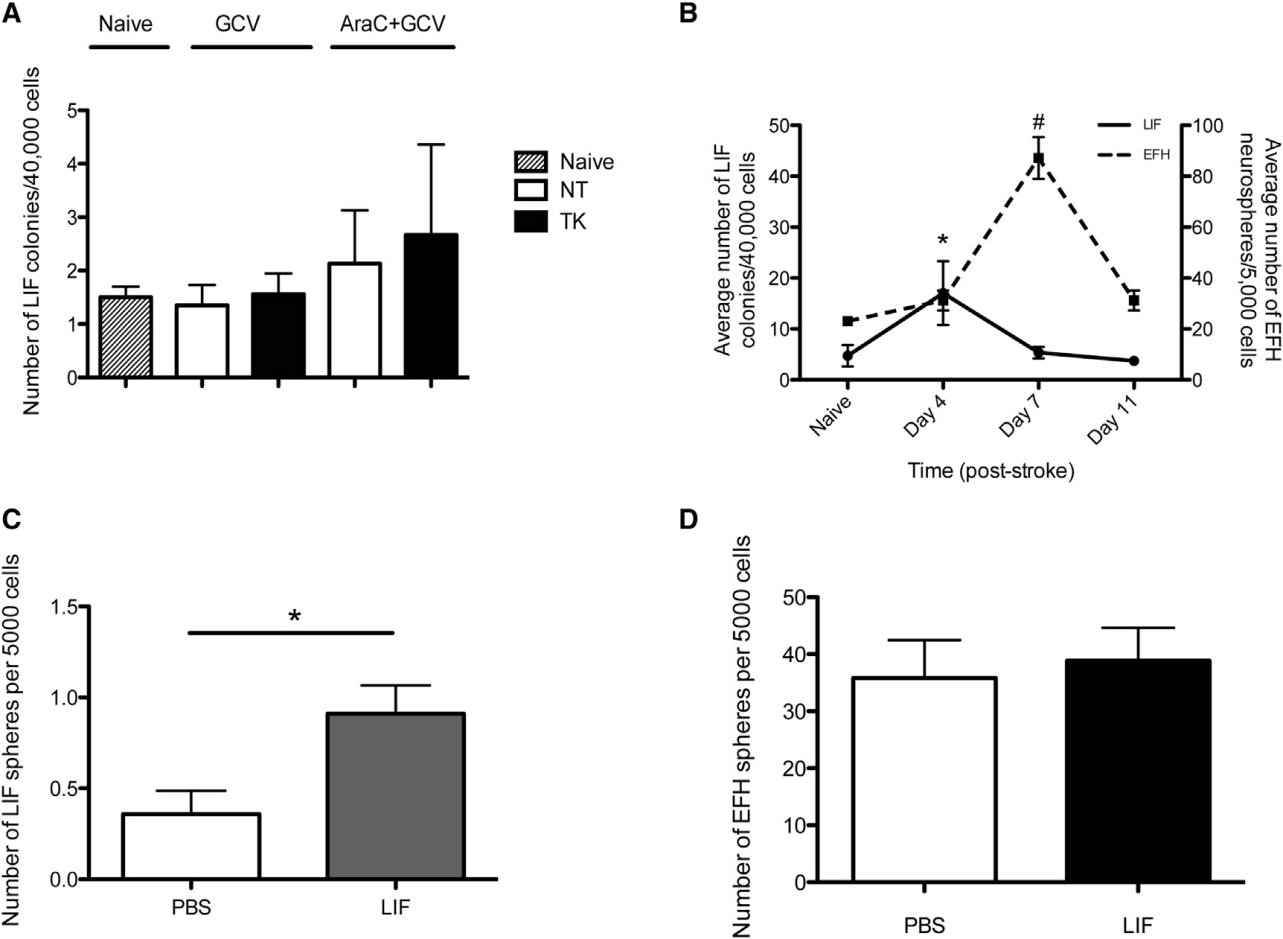
Numbers of Adult-Derived pNSCs Can Be Increased by Injury or LIF Infusion (A) Numbers of LIF colonies isolated after GCV infusion (3 days) (n ≥ 22 mice/group) or AraC and GCV infusion (n = 5 mice/group). (B) Stroke-injured mice generated significantly more LIF colonies at 4 days poststroke and returned to control values at day 11 (n = 6–12 mice/group/time). Significance determined by one-way ANOVA with post hoc Bonferroni test, ^∗^p < 0.05 from LIF naive to LIF day 4, #p < 0.05 from EFH naive to EFH day 7. The number of EFH neurospheres significantly increased at 7 days poststroke (n > 3 mice/group). (C and D) Four-day LIF infusion resulted in a significant increase in (C) LIF colonies, but not (D) EFH neurospheres (n = 6 mice/group). Significance determined by Student’s t test (p < 0.05) unless otherwise stated. Data are shown as mean ± SEM. See also Figure S5.

The AdpNSC population comprise a very rare population of cells with an average of 5.6 ± 2.5 colonies per brain from naive controls, with similar numbers from GFAP-TK mice when grown in the presence or absence of GCV, and from AraC- and GCV-treated GFAP-TK and NT mice (Figure 6A). The lack of expansion in numbers suggests that AraC and GCV treatment results in asymmetric divisions of the AdpNSC to repopulate the GFAP^+^ NSCs during regeneration of the SE, thereby maintaining their absolute numbers. We reasoned that an expansion of this rare AdpNSC population might occur in an injury model such as stroke, where there is no depletion in the GFAP^+^ adult NSC fraction (Zhang et al., 2004). We previously demonstrated that the pial vessel disruption (PVD) model of stroke results in increased numbers of EFH neurospheres poststroke (Erlandsson et al., 2011). We used the PVD model to determine whether LIF colony formation was also increased after injury. Mice received a stroke lesion on day 0, and the number of AdpNSC LIF colonies was examined at 4, 7, and 11 days poststroke. We observed a significant 5.4-fold increase in the number of AdpNSC LIF colonies at day 4, with a return to control numbers by day 7 poststroke. Stroke also resulted in a significant 3.8-fold increase in the number of EFH neurospheres at day 7 poststroke (Figure 6B). These data suggest that AdpNSCs are sensitive to environmental cues that result after injury. Most interestingly, the AdpNSCs expansion occurred more rapidly and prior to the definitive GFAP^+^ NSC expansion, supporting the hypothesis that the AdpNSC resides upstream of the GFAP^+^ adult NSCs.

Many types of injuries to the nervous system are accompanied by a rapid and transient increase in LIF expression; hence, we asked whether increased LIF signaling played a role in the activation of AdpNSCs in our stroke model (Banner et al., 1997; Suzuki et al., 2005; Bauer et al., 2003). Intraventricular infusion of LIF for 4 days resulted in a significant 2.5-fold increase in the number of LIF colonies from adult mice (Figure 6C), with no increase in the numbers of EFH-responsive neurospheres (Figure 6D). These data indicate that the AdpNSC pool expands in response to LIF in vivo.

## Discussion

**Figure 7:**
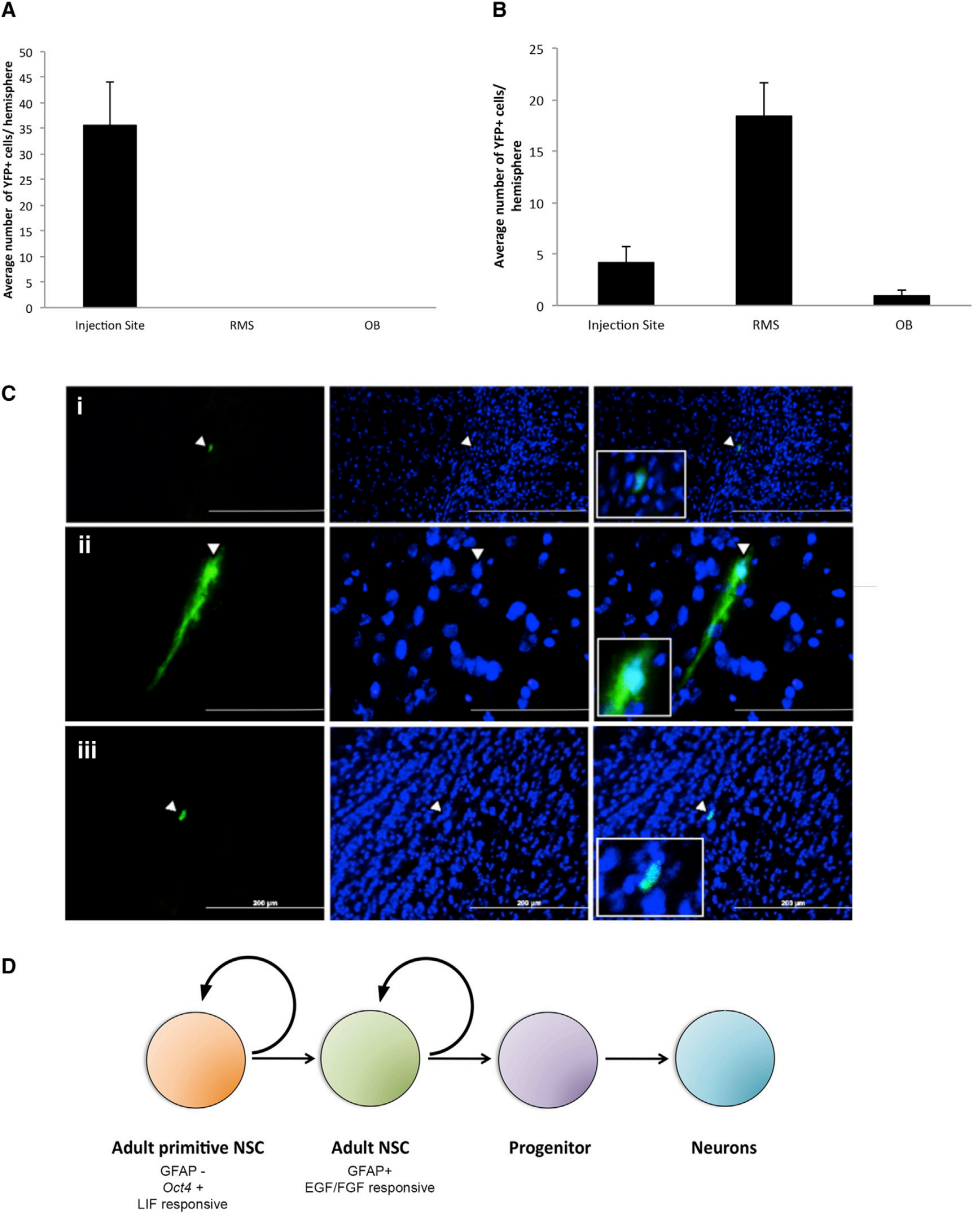
In Vivo Lineage Analysis (A) Distribution of YFP^+^ cells at 48 hr posttransplant (n = 4 mice). (B) Distribution of YFP^+^ cells at 14 days posttransplant (n = 7 mice). (C) Representative images of cells (i and ii) along the RMS and (iii) in the OB after 14 days. Insets show higher magnification of YFP^+^ cells. (D) Adult pNSCs are GFAP^−^, responsive to LIF, and express the pluripotency marker *Oct4*. The self-renewing, multipotent AdpNSCs give rise to EFH-responsive, definitive adult NSCs. The definitive adult NSCs are neurogenic in the adult brain. Data are shown as mean ± SEM. Scale bars represent (200 μm (Ci), 70 μm (Cii), and 200 μm (Ciii). See also Figure S6.

Our results demonstrate the existence of a rare population of pNSCs in the adult brain that express *Oct4* and have the ability to integrate into the ICM of blastocyst chimeras. This LIF-responsive population acts as a reserve pool capable of repopulating the neural lineage in the SE in vivo. Similar to its embryonic counterpart, the AdpNSC is a GFAP^−^, LIFR^+^ cell from the periventricular region of the brain. The inability to permanently ablate GFAP^+^ neurosphere-forming cells after a complete initial loss suggests that the AdpNSC is upstream of the GFAP^+^ adult NSC (Figure 7D). This lineage relationship is supported by both in vitro and in vivo findings. In vitro, the passaging of the LIF colonies into standard adult neurosphere conditions (EFH) revealed that LIF colonies gave rise to GFAP^+^, neurosphere-forming cells. In vivo, LIF-colony-derived cells devoid of GFAP^+^ cells at the time of transplantation were able to migrate along the RMS and contribute to neurogenesis. Further, we showed that Floxed *Oct4*-*Sox1*Cre mice that completely lacked AdpNSCs were unable to repopulate the GFAP^+^, neurosphere-forming NSCs after ablation in vivo. Hence, based on the studies described herein, we propose that the AdpNSC proliferates in response to injury and gives rise to GFAP^+^ adult NSCs that repopulate the SE after their ablation in vivo.

Several pieces of evidence indicate that GFAP^+^ NSCs can be ablated completely in vivo after GCV treatment in GFAP-TK mice, and that they are subsequently reconstituted from a GFAP^−^ cell in the NSC lineage. First, zero neurosphere-forming cells were observed immediately after GCV treatment for 21 days, or after AraC and GCV treatment in GFAP-TK mice. Second, GCV remains toxic to GFAP^+^ cells for periods of time exceeding the infusion times used here; however, GFAP^+^ NSC-derived clonal neurospheres inevitably returned in vitro and their proliferating progeny returned in vivo. Third, the neurospheres that returned at longer survival times after GCV infusion were lost when GCV was added in vitro in all instances, and therefore must have been derived from GFAP^+^ NSCs. Fourth, the observation that EGF alone did not support neurosphere formation immediately after GCV infusion indicates that the neurospheres were not derived from transit-amplifying cells. Finally, the finding that the kinetics of the return of GFAP^+^ NSCs was dramatically different in paradigms that did not completely eliminate neurosphere formation (i.e., AraC treatment alone) versus when there was a complete loss of GFAP^+^ NSCs (AraC and GCV treatment) suggests that different cell sources may be responsible for repopulation of the SE. Together, these data support the hypothesis that the GFAP^−^ (resistant to GCV treatment) AdpNSC is able to repopulate the GFAP^+^ NSC population.

The numbers of GFAP^+^ NSCs did not return to control levels at even the longest survival times examined (approximately 1 month after AraC and GCV treatment). One possible explanation for this is that we simply did not wait long enough to observe the complete return to control values. Alternatively, the AdpNSCs may only be able to proliferate a limited number of times, as has been suggested by studies showing that serial transplantation of hematopoietic stem cells for repopulation was limited to five to seven rounds (Harrison et al., 1978; Harrison and Astle, 1982). The fact that the AdpNSCs were not killed by the AraC treatment indicates that the cell has a relatively long cell-cycle time, in line with studies that identified slowly cycling cells in other systems (Fuchs, 2009). Indeed, reports suggest that the blood system has a population of long-term label-retaining cells that proliferate only once every 4–5 months, translating into approximately five divisions in the life of an animal (Foudi et al., 2009; Wilson et al., 2008). This dormancy may be important for maintaining “stemness” or it may be a property of the “master” stem cells that are activated in times of stress or injury (Fuchs, 2009). The existence of an AdpNSC that can be activated in response to injury suggests that these slowing cycling AdpNSCs are called upon to divide only rarely to generate the GFAP^+^ adult NSCs that are responsible for maintaining adult neurogenesis under baseline conditions.

We propose that the AdpNSC is CD133^−^ based on the lack of colocalization of *Oct4* with β-catenin; however, others have proposed the presence of a stem cell-like CD133^+^ cell in the adult brain. In a previous study, it was reported that quiescent *CD133*-expressing, ciliated ependymal cells responded to injury by entering into the cell cycle and contributing cells to tissue regeneration (Carlén et al., 2009). The activated ependymal cells were unable to self-renew to maintain their population, suggesting that they may represent a nonstem cell response to injury (Carlén et al., 2009). The *CD133*^+^ ependymal cells’ inability to self-renew makes them distinctly different from the AdpNSCs, which are able to self-renew in vivo, as illustrated by their expansion in number after LIF infusion and stroke. *CD133* has been shown to be present on the apical surface of GFAP^+^ adult NSCs (Mirzadeh et al., 2008), and we also detect *CD133* in LIF colonies; however, we suggest that this CD133 expression is due to the presence of GFAP^+^ progeny within the LIF colony. Further, we do not see colocalization of *Oct4* with β-catenin, which has been shown to identify ependymal cells in whole-mount sections of the lateral ventricle periventricular region (Mirzadeh et al., 2008). Taken together, these findings suggest that AdpNSCs do not correspond with the *CD133*^+^ ependymal cells previously studied.

In conclusion, we have identified a rare pNSC that is present in the adult brain. The adult-derived pNSC has characteristics of pluripotency, including *Oct4* expression and the ability to integrate into the ICM of blastocyst chimeras. Similar to pNSCs during development, the progeny of the AdpNSC include the GFAP^+^, neurogenic, neurosphere-forming, type B NSCs that are present in the adult SE. We propose that this LIFR^+^ pNSC may be an additional target for the development of regenerative medicine strategies in the adult CNS.

## Experimental Procedures

### Animals

Animals were maintained in the Department of Comparative Medicine at the University of Toronto in accordance with institutional guidelines.

### Cell Culture

Mice were sacrificed by cervical dislocation. Brains were dissected and the periventricular region was cultured as previously described (Chiasson et al., 1999). For neurosphere assays, cells were plated in serum-free media in EFH or in LIF alone. For adherent colonies, cells were plated on MEFs in standard ESC media supplemented with LIF and passaged once weekly as previously described (Tropepe et al., 2001). EFH neurospheres or LIF colonies were counted at 7–10 days in vitro.

### Surgery

For all surgical procedures, animals were anesthetized with 1%–5% isoflurane and injected with ketoprofen (5 mg/kg). Stroke was induced by removing the skull and dura in the region bound by −0.5 mm, +2.5 mm (AP) and +0.5 mm, 3.0 mm (M/L) relative to bregma. A saline-soaked cotton swab was used to remove pial vessels. GCV (200 μM) and 2% AraC were infused via a mini osmotic pump (Alzet 1007D) with cannulas placed at 0.2 mm A/P, 0.7 mm M/L, and 2.5 mm D/V relative to bregma. Transplanted animals received 1 μl of 800 cells injected at 0.5 mm M/L, 1.5 mm A/P, and 2.5 mm D/V relative to bregma.

### qPCR

ESCs, LIF colonies, and EFH neurospheres were collected into Buffer RLT (QIAGEN) with β-mercapthenol. Samples were processed according to the manufacturer’s directions using the RNeasy Micro Kit (QIAGEN), including treatment with the RNase-free DNase Set (QIAGEN). cDNA synthesis was carried out with Superscript III First Strand Synthesis System (Invitrogen). qPCR was carried out on a 7900HT Fast Real-Time PCR System (Applied Biosystems). Cycling conditions consisted of initial activation (2 min at 50°C and then 10 min at 95°C), followed by 40 cycles of 15 s at 95°C and 1 min at 60°C, followed by 15 s at 95°C, 15 s at 60°C, and 15 s at 95°C.

### Tissue Preparation and Immunohistochemistry

Mice were sacrificed with an overdose of sodium pentobarbital and perfused transcardially with ice-cold PBS followed by 4% paraformaldehyde, postfixed, and cryoprotected in 20% sucrose. For immunohistochemistry, sections were rehydrated with PBS and membranes were permeabilized with 0.3% Triton-X in PBS for 20 min at room temperature. For BrdU imaging, DNA was denatured with 1 N HCl at 65°C for 30 min. Sections were blocked with 10% normal goat serum or 10% normal donkey serum (Sigma) in PBS for 1 hr at room temperature before incubation with primary antibodies at 4°C overnight, followed by incubation of secondary antibodies for 1 hr at 37°C. Whole-mount sections were derived from *Oct4*-GFP adult mice as previously described (Mirzadeh et al., 2010). Staining was visualized on an AxioVision Zeiss UV microscope and a Nikon 200 microscope, or an Olympus Fluroview FV1000 confocal laser scanning microscope.

### Statistics

Data are represented as mean ± SEM unless otherwise stated. Statistical analysis was performed by GraphPad Prism 5 (GraphPad Software) using ANOVA with Bonferroni’s multiple comparison test or Student’s t test unless otherwise stated.

## Author Contributions

N.S. collected, assembled, analyzed, and interpreted data and gave final approval of the manuscript. R.L., W.X., K.L.R., and F.Y. collected data. R.L. and W.X. contributed equally to the work. D.v.d.K. conceived and designed study, provided financial support, analyzed and interpreted data, and gave final approval of the manuscript. C.M.M. conceived and designed study, provided financial support, analyzed, interpreted, and assembled data, and wrote and gave final approval of the manuscript.

## Supplemental Information

Document S1. Figures S1–S6 and Supplemental Experimental Procedures

Movie S1. Oct4^+^ Cells in the Periventricular Region of an Adult Mouse, Related to Figure 2 Confocal video of *Oct4* positive cell (green) in whole-mount sections. *Z* stack of five slices, 1 μm thick each.

Document S2. Article plus Supplemental Information
