# Supplementary material for: Primitive Neural Stem Cells in the Adult Mammalian Brain Give Rise to GFAP-Expressing Neural Stem Cells
Source: Stem Cell Reports. 2014 May 22;2(6):810–24. doi: 10.1016/j.stemcr.2014.04.008 (PMC4050350; doi:10.1016/j.stemcr.2014.04.008)
Supplement: Document S1. Figures S1–S6 and Supplemental Experimental Procedures [file mmc1.pdf]

Stem Cell Reports, Volume *2*

Supplemental Information

Primitive Neural Stem Cells in the Adult Mammalian Brain Give Rise to GFAP-Expressing Neural Stem Cells

Nadia Sachewsky, Rachel Leeder, Wenjun Xu, Keeley L. Rose, Fenggang Yu, Derek van der Kooy, and Cindi M. Morshead

SUPPLEMENTAL INFORMATION

Figure S1 (related to Figure 1). The periventricular region contains proliferating LIFR$^+$ cells *in vivo*. Confocal images showing the presence of LIFR$^+$ cells within the SE in naïve control mice. (A) A subpopulation of SE LIFR$^+$ cells (green) are proliferative (Ki67$^+$, red). (B) After AraC+GCV infusions (7 days each), Ki67$^+$ cells (red) are seen in the SE of TK mice. A subpopulation of Ki67$^+$ cells are LIFR$^+$ (arrows). (C) SOX2 (red) is widely expressed in both the ependyma and SE and colocalizes with LIFR (arrows). (D) Some LIFR$^+$ cells also express GFAP (red) in the subependyma of AraC+GCV treated TK mice (arrows). Scale bar=15 μm. Blue = Hoechst labeled nuclei.

Video S1 (related to Figure 2). Confocal video of *Oct4* positive cell (green) in wholemount sections. Z-Stack of 5 slices, 1 μm thick each.

Figure S2 (related to Figure 3). Expression profile of AdpNSCs. (A) Gene expression profile of AdpNSC derived LIF colonies (orange bars) and EFH neurospheres (green bars) compared to ES cells assayed by quantitative qPCR (n=3 independent replicates per group). Significance between LIF colonies and EFH neurospheres is denoted by (*) and significant difference to ES cells is denoted by (#), calculated based on the log value by 2-way ANOVA with Bonferroni post-hoc test ($p<0.05$). (B) Immunohistochemical analysis of wholemount sections from *Oct4*-GFP mice reveal (i) *Oct4*-GFP (arrows) does not co-localize with GFAP but does co-localize with (ii) LIF receptor. (iii) *Oct4*-GFP does not co-localize with β-catenin. Scalebars = 10μm. (C) Representative images of FACS analysis on (i) periventricular cells derived from naïve adult

Oct4-GFP animals as well as a (ii) positive control using *actin*-GFP mice, (iii) negative control using CD1 mice, and (iv) transgenic control using *Oct4*-GFP cortex to set appropriate gates (n=2 independent experiments). Data represent mean +/- SEM.

Figure S3 (related to Figure 4). The effects of GCV *in vitro* and *in vivo*. (A) Exposure to 20 μM GCV *in vitro* does not affect the size of neurospheres from NT littermate controls (shown) or C57Bl/6 controls (data not shown) (n=3 independent experiments). (B) $GFAP^-$ type C progenitor cells are not responsible for the return of neurospheres *in vitro* as EGF responsive neurospheres could be isolated from untreated control mice (NT, n=6), saline-infused mice (S, n=3), and NT mice that received 7d GCV (n=3), AraC + GCV (n=2), or 21d GCV (n=2). EGF-responsive neurospheres could not be isolated from TK mice following 7 day GCV (n=4), AraC + GCV (n=3), or 21 day GCV (n=3) exposure in the absence of GCV *in vitro*. (C) 2 day GCV infusion (n $\geq$ 4 mice/group) followed by sacrifice at various times post-infusion results in a return of EFH neurosphere formation over time. Data represent mean +/- SEM.

Figure S4 (related to Figure 5). Proliferating $GFAP^+$ cells returned with longer survival times following AraC+GCV treatment. (i, ii) Representative confocal images showing $GFAP^+$ and $Ki67^+$ cells in the SE of TK (i) and NT (ii) mice immediately following AraC+GCV infusions (7 days each) (day 14). (iii, iv) By day 42, post-infusion $GFAP^+/Ki67^+$ SE cells are observed in both TK and NT mice. Scalebars = 10μm.

Figure S5 (related to Figure 6). (A) An initial loss of $BrdU^+$ cells in the subependyma (SE) and rostral migratory stream (RMS) of TK mice is followed by a return to control levels by day 42. Data represents mean ± s.e.m. (n=3-4 mice per group, 5 sections/mouse per region). *$p<0.05$. (B) GCV remains toxic to dividing $GFAP^+$ cells at least 10 days after exposure. Astrocyte cultures generated from TK and NT mice were exposed to GCV for 12 hours, re-plated in fresh media and maintained as monolayer cultures for up to 21 days. On day 5, 8, 10 and 21 after GCV exposure, cells were induced to divide by passaging and plating in standard neurosphere conditions (EFH). TK cell cultures failed to generate neurospheres up to 10 days post GCV pulse. Even at 21 days post GCV exposure, only rare neurospheres formed from TK cultures (5 versus 746 neurospheres per 360,000 cells plated from TK versus NT cultures respectively). (n ≧ 2 independent experiments/timepoint). (C) Representative pictures of cultures from TK and NT cultures. At all times examined, the GCV exposed NT and TK cultures had viable, healthy cells in the standard EFH conditions. Anti-GFAP immunostaining shows astrocytes in TK and NT derived cultures at day 10 prior to dissociation and plating in the EFH. Data represent mean +/- SEM. Scale bars: all TK cultures 200 μm; NT cultures at days 5 and 10, 100 μm, day 8, 200 μm. GFAP staining, 200 μm.

Figure S6 (related to Figure 7). YFP-GFAPtk derived colonies for transplantation. (A) YFP-GFAPtk (YFP-TK) mice generate YFP positive LIF colonies in the presence of GCV similar to non-transgenic littermate controls (YFP-NT) grown in GCV. (B) The frequency of YFP-TK derived LIF colonies in the presence of GCV is not different from YFP-NT littermate controls; however, YFP-TK LIF colonies were smaller in size (n=3 mice/genotype). (C) Only YFP-NT derived LIF colonies grown in GCV passage into EFH conditions indicating that the LIF

colonies YFP-TK derived LIF colonies do not contain GFAP+ neurosphere forming cells (n=3 animals per genotype). (D) Representative images of cells (i) migrating, (ii-iii) differentiating, and (iv) expressing the neuroblast marker DCX at 14 days post-transplant. Data represent mean +/- SEM. Scalebars = 20μm.

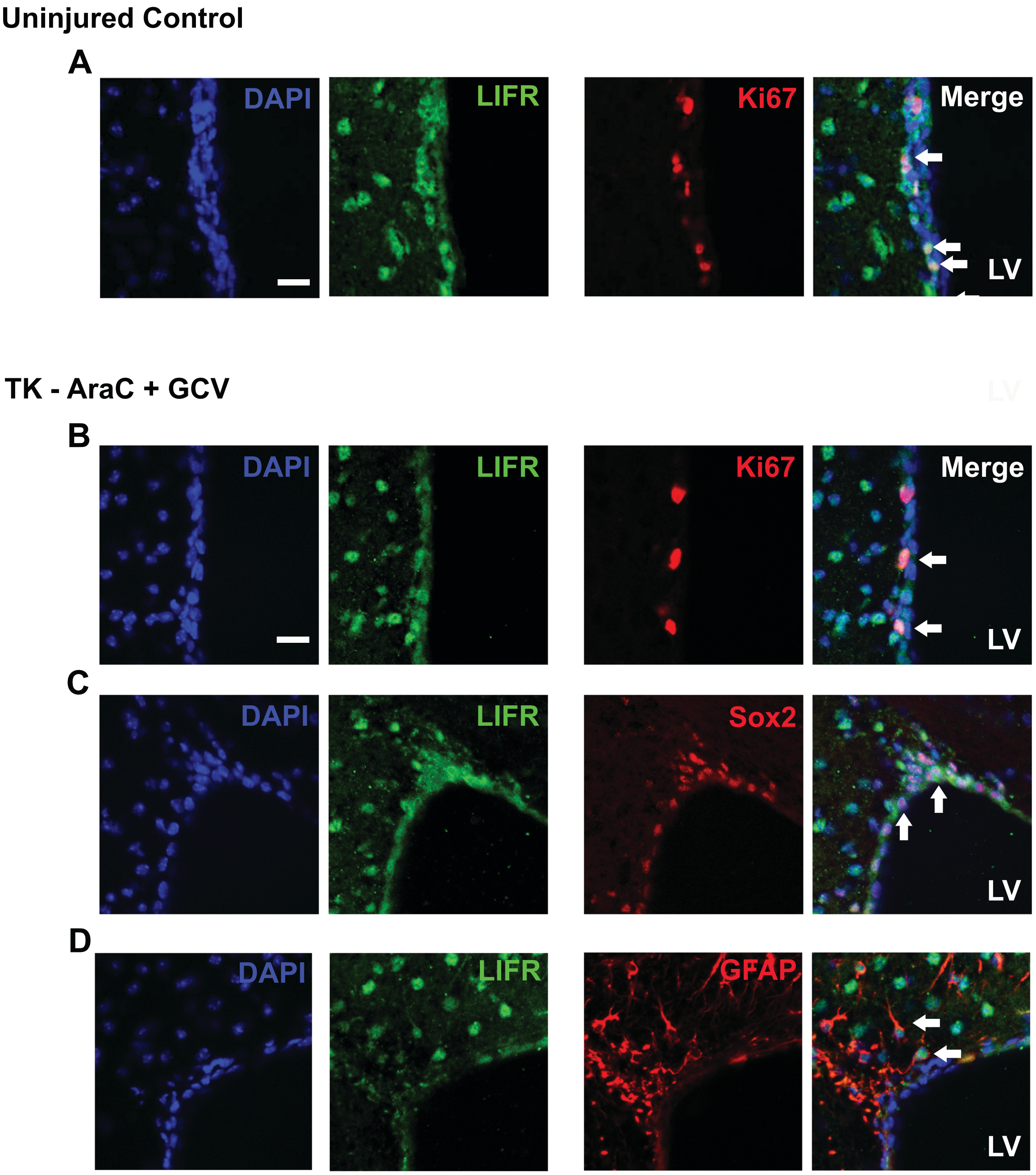

Supplemental Figure 1

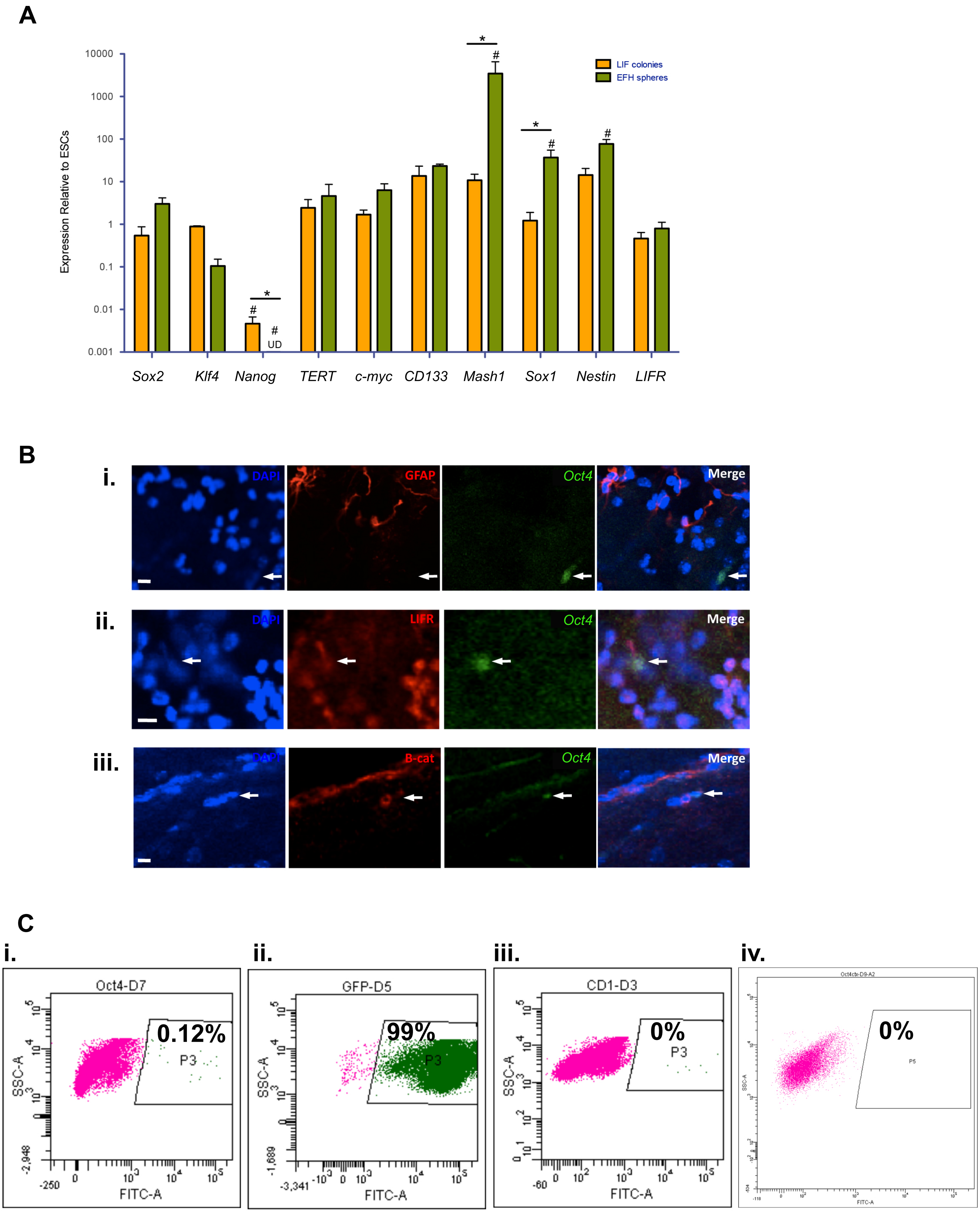

Supplemental Figure 2

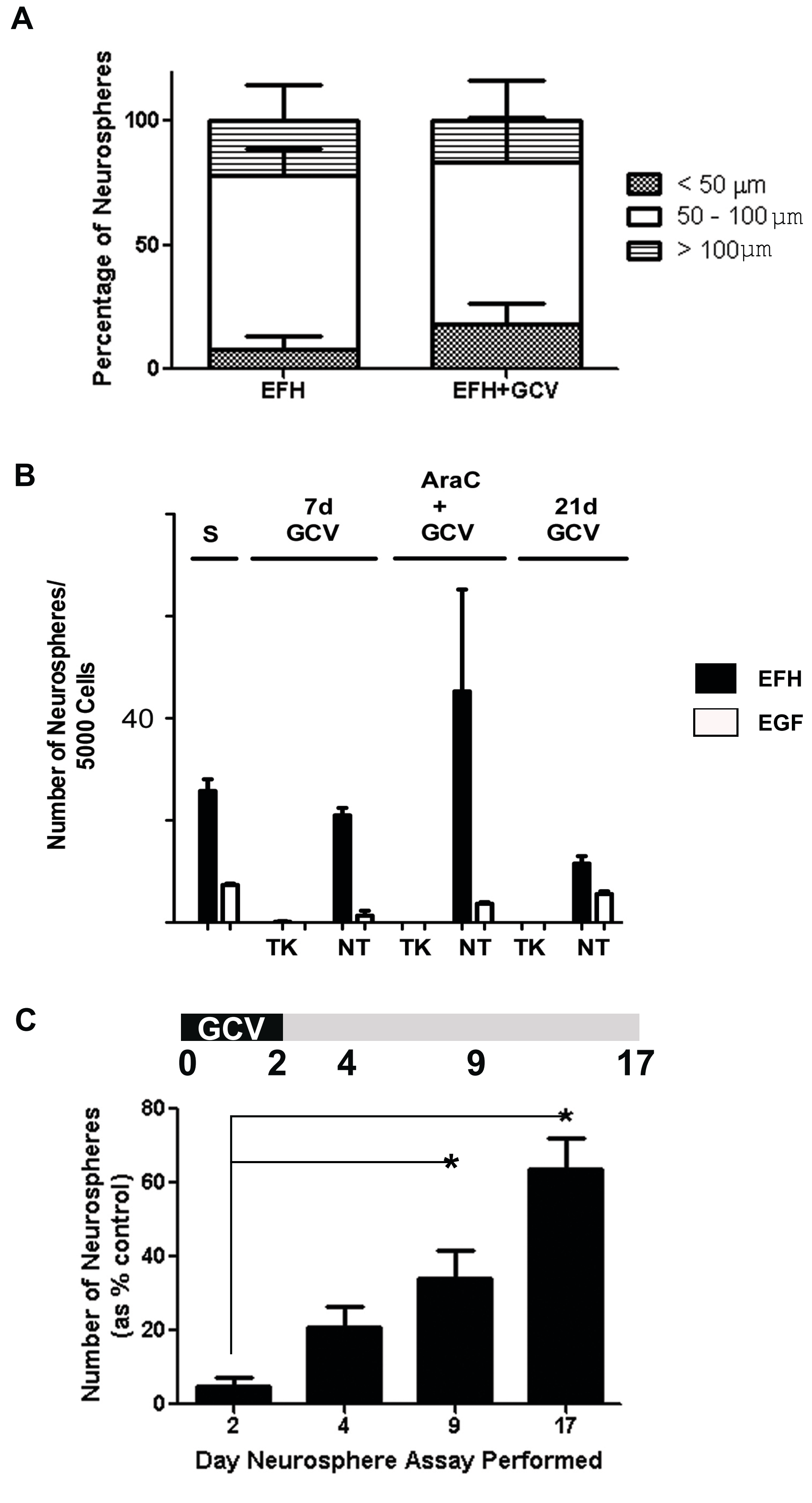

Supplemental Figure 3

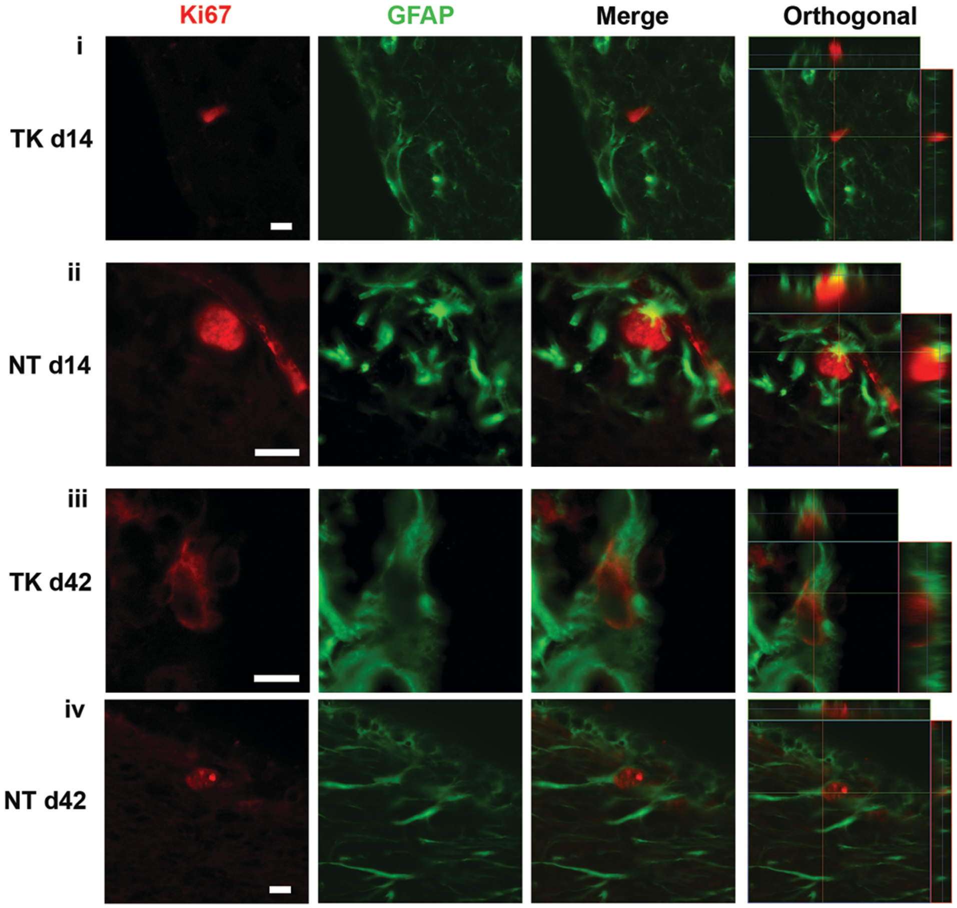

Supplemental Figure 4

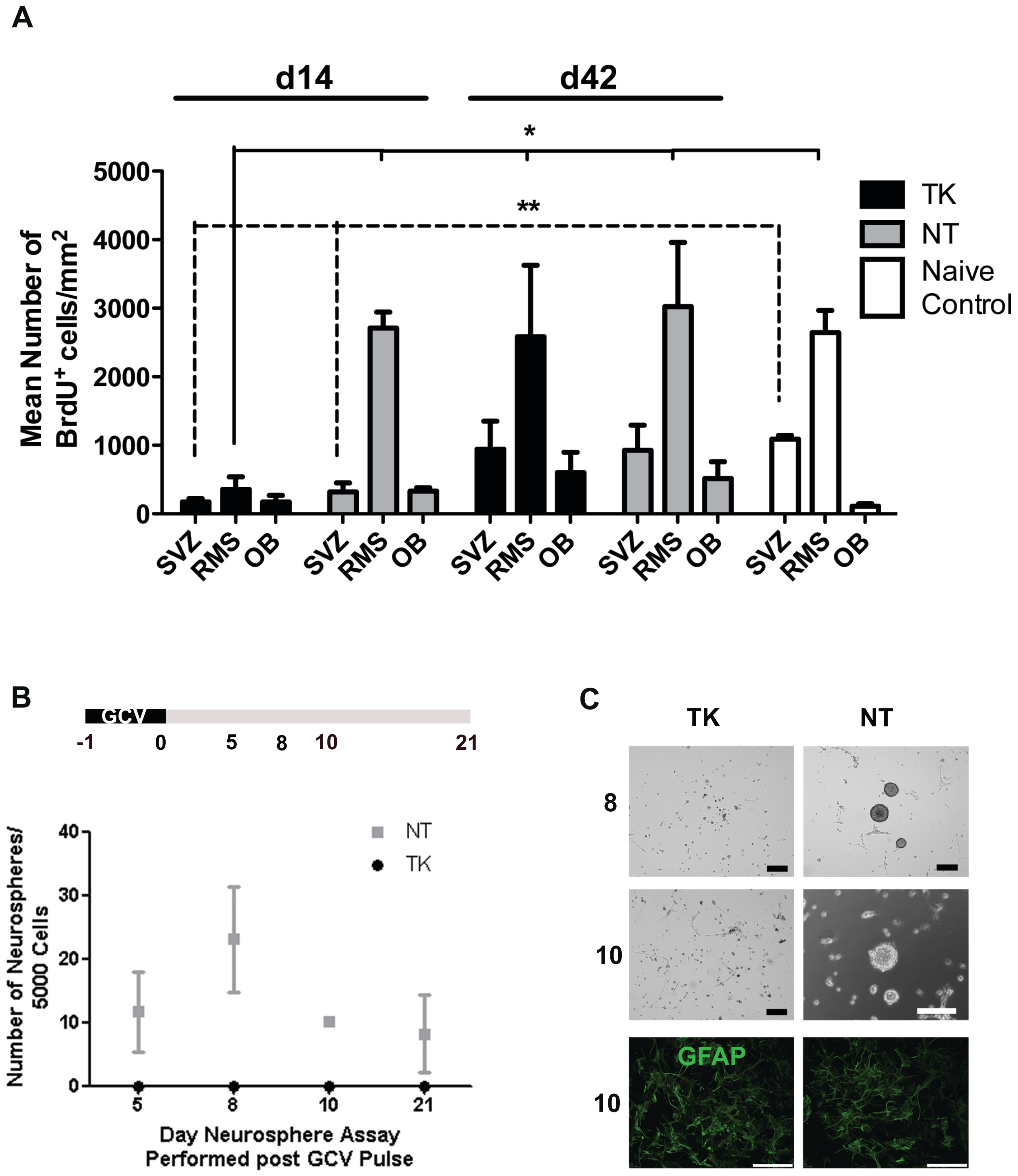

Supplemental Figure 5

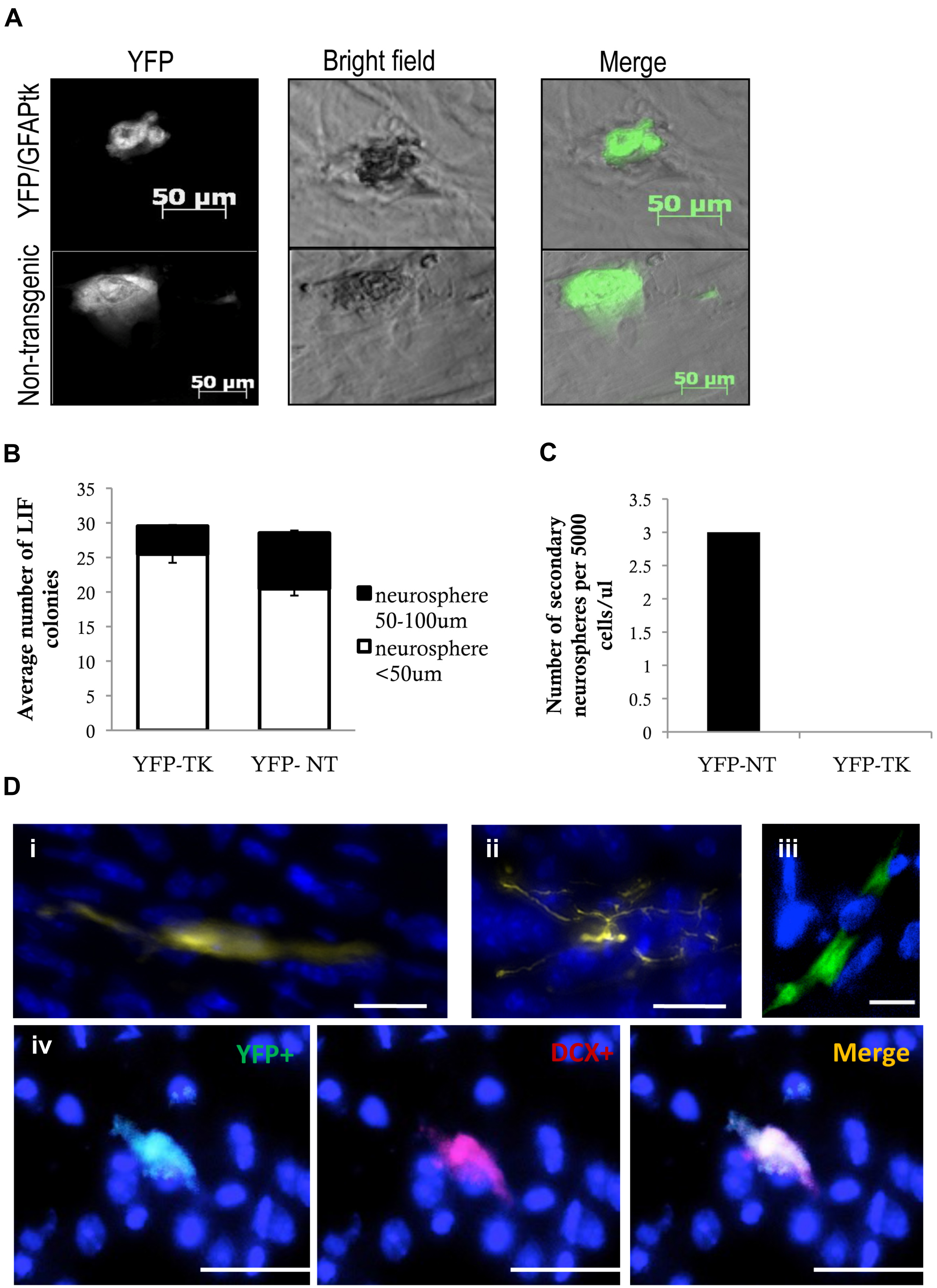

Supplemental Figure 6

SUPPLEMENTAL EXPERIMENTAL PROCEDURES

Animals. GFAP-TK Line 7.1 mice (a kind gift from Dr. M. Sofroniew, UCLA), *Oct4*-GFP mice (a kind gift from Dr. A Nagy, University of Toronto), Floxed *Oct4* mice (a kind gift from Dr. A. Tomlin, UPenn), Sox1-Cre mice (a kind gift from Dr. S. Nishikawa, Japan), YFP reporter mice, adult C57Bl/6 and CD1 (Charles River) mice, and *Oct4*-neo (Jackson Labs) were maintained in the Department of Comparative Medicine at the University of Toronto in accordance with institutional guidelines. GFAP-TK Line 7.1 is well characterized and the transgenic protein alone is not harmful to mammalian cells. Previous analyses at the single cell level on GFAP-TK line 7.1 mice showed 100% of TK expressing cells were also $GFAP^+$ in the SE (Garcia et al., 2004; Morshead et al., 2003). GFAP-TK and *Oct4*-neo mice were genotyped employing primer sequences from original source.

Neurosphere Assay. Mice were sacrificed by cervical dislocation. Brains were dissected and the periventricular region was cultured as previously described (Chiasson et al., 1999). For negative control samples, cortical and striatal tissue was sampled from brains and processed at the same time and under identical conditions as periventricular tissue. Cells were plated at clonal density (Coles-Takabe et al., 2008) (10 cells/µL) in 24 well culture plates (Nunclon, 500 µL/well). Standard neurosphere culture conditions consisted of serum free media (SFM) supplemented with epidermal growth factor (EGF, 20 ng/mL; Sigma), basic fibroblast growth factor (bFGF, 10 ng/mL; Sigma) and heparin (2 µg/mL; Sigma). Some cultures were grown in EGF only, LIF only (10.2 ng/mL) (Tropepe et al., 2001). GFAP-TK and controls were plated in the presence or absence of GCV (Sigma, G2536). Cultures derived from *Oct4*-neo mice or controls were plated in the presence or absence of neomycin (G418; 800 µg/ml, Sigma). EFH neurospheres or LIF colonies were counted at 7-10 days *in vitro*.

Self-renewal and differentiation assays. For self-renewal assays, individual neurospheres or colonies were collected and placed in an Eppendorf tube containing 200 μl SFM + appropriate growth factors, triturated, and transferred into 300 µL of media in a 24 well plate to a total volume of 500μl per well. The numbers of neurospheres or colonies was assayed after 7-10 days *in vitro*. For differentiation, individual neurospheres or colonies were plated on Matrigel (1:24 dilution in SFM) in a 24 well plate in SFM with 1-5% fetal bovine serum. After 7-9 days *in vitro*, cells were fixed with 4% PFA for 20 minutes at RT, then washed with PBS.

Stroke. CD1 or YFP transgenic mice were anaesthetized using isofluorane and injected with Ketoprofen (5mg/kg). A rectangular hole was drilled into the frontal and parietal bones running from 0.5 mm posterior to 2.5 anterior to the bregma and running from 0.5 to 3.0 mm lateral from the midline. The dura was removed and a sterile saline soaked cotton swab was used to wipe the blood vessels from the cortical surface. At day 4, 7 and 11 days following stroke the mice were killed by cervical dislocation and the brains were dissected and plated for LIF colony formation.

ES and LIF colonies on feeders. Cells were maintained on mouse embryonic fibroblasts (MEFs) in standard ES media supplemented with LIF and passaged once weekly as previously described (Tropepe et al., 2001). ES cell colonies were established from YFP mice and maintained continuously. Adult forebrain cells were derived from YFP or *Oct4*-GFP mice 4 days following stroke and maintained identically to ES cells. After 2-12 passages, the adult derived colonies from YFP mice were used for chimera assay by individual selection of colonies.

GCV and AraC infusions. Mice were anaesthetized with 5% isoflurane, injected with Ketoprofen (5 mg/kg), and a mini osmotic pump (Alzet 1007D, Direct Corp.) was implanted into the right lateral ventricle (+0.2 mm anterior/posterior, +0.7 mm medial/lateral, and 2.5 mm below surface of the skull, relative to bregma). The pump concentration of GCV was 200 μM in sterile PBS. Intraventricular infusion of 2% AraC (Sigma, C1768) in sterile 0.9% saline followed the same protocol.

BrdU labeling. Mice received 3 injections of BrdU (60 mg/kg i.p.; Sigma), 1 every 3 hours, one day prior to sacrifice. $BrdU^+$ cells from each brain region were counted blinded to genotype and from a minimum 3 sections per region per mouse.

Transplantations. Wild-type CD1 adult mice (8-10 weeks) were anesthetized with isoflurane and administered ketoprophen (5mg/kg). Transplant co-ordinates were determined from Bregma at 0.5 lateral, 1.5 anterior, and 2.5 ventral. 1μl of single cell suspension containing 800 YFP-GFAPtk LIF colony-derived cells that had been grown in the presence of GCV for 2 weeks was injected at this location. Animals were allowed to recover and were sacrificed by intracardial perfusions of 4% PFA at 4 hours post-transplantation, 7 days, or 14 days. Brains were post-fixed and cryoprotected in 4% PFA in 20% sucrose overnight and sectioned for YFP cell counts.

qPCR. ES cells and LIF colonies were trypsinized and re-plated. After 30 minutes, the fibroblasts adhered to the plate, and the colonies, which remained suspended, were collected with the media, centrifuged and resuspended in Buffer RLT with β-mercapthenol. EFH neurospheres and pNSC neurospheres were individually picked and collected into Buffer RLT with β-mercapthenol. Fresh tissue was derived from adult mice cortex and periventricular region. Samples were processed as per manufacturer's directions using the RNeasy Micro Kit (Qiagen, Mississauga, ON), including treatment with the RNase-free DNase Set (Qiagen). cDNA synthesis was carried out with Superscript III First Strand Synthesis System (Invitrogen, Carlsbad, CA). QPCR was carried out on a 7900HT Fast Real-Time PCR System (Applied Biosystems). Cycling conditions consisted of initial activation (2 min at 50ºC, then 10 min at 95ºC), followed by 40 cycles of 15s at 95ºC, 1 min at 60ºC, followed by 15s at 95ºC, 15s at 60ºC and 15s at 95ºC. The data were converted into threshold cycles using SDS 2.3 (Applied Biosystems), normalized relative to ES cells and expressed as logarithmic values using Microsoft Excel, and plotted with Graphpad Prism (Graphpad Software, Inc., LaJolla, CA). Taqman Probes: GAPDH (Mm03302249_g1) and *Oct4*/Pou5fl (Mm03053917_g1) Sox2 (Mm03053810_s1), Klf4 (Mm00516104_m1), nanog (Mm02019550_s1), TERT (Mm01352136_m1), c-myc (Mm00451051_g1), CD133 (Mm00477115_m1),

Mash1/Ascl1 (Mm03058063_m1), Sox1 (Mm00486299_s1), Nestin (Mm00450205_m1), LIF-R (Mm00442942_m1) (Applied Biosystems).

Chimera Assay. Morulas were derived from CD1 mice as previously described (Karpowicz et al., 2007) and aggregated with 8-10 cell clusters from various cell types: ES colonies on MEFs, LIF colonies on MEFs, or free-floating EFH neurospheres. The cells and morulas were aggregated overnight at 37ºC in individual aggregation wells. Images were captured during blastoceol cavity formation on a Zeiss Observer D1 with AxioCam MRm.

Astrocyte monolayers. Astrocyte monolayer cultures were generated from neonatal mouse cortex as previously described (Imura et al., 2003). Cells were mechanically dissociated and passed through 40 μm mesh filters and plated in 6 well plates or T25 tissue culture flasks at a density of 50 000 cells/cm^2 in DMEM/F12 (Gibco, Invitrogen) supplemented with 10% FBS. Upon reaching confluence, monolayers were shaken overnight at 200 rpm to remove macrophages and oligodendrocytes, then monolayers were pulsed with final concentration of 20-30 μM GCV for 12 hours. GCV was washed out thoroughly (3 x 10 minute washes) with 1xPBS and media was replaced daily. At 5, 8, 10 and 21 days post-GCV exposure, monolayers were trypsinized and replated in standard neurosphere conditions (EFH).

Tissue preparation and immunohistochemistry. Mice were sacrificed with an overdose of sodium pentobarbital and perfused transcardially with ice cold PBS followed by 4% PFA. Brains were post-fixed overnight at 4ºC then cryoprotected in 20% sucrose until sectioning. Coronal sections (14 μm) were cut on a cryostat (-20 ºC) from the rostral tip of the anterior commissure through the olfactory bulbs. Every 10th section from each brain region (≥5 sections per slide) per region was examined (lateral ventricle, RMS and OB). For immunohistochemistry, sections re-hydrated with PBS and membranes were permeabilized with 0.3% Triton-X in PBS for 20 minutes at RT. For BrdU imaging, DNA was denatured with 1N HCl at 65ºC for 30 minutes. Sections were blocked with 10% NGS (normal goat serum) or 10% NDS (normal donkey serum (Sigma)) in PBS for 1 hour at RT before incubation with primary antibodies at 4ºC overnight. The primary antibodies were rat anti-BrdU monoclonal (Abcam, ab6326 1:100), mouse anti-PSA-NCAM monoclonal (Millipore, MAB5324 1:500), mouse anti-CD133 monoclonal (eBioscience, 14-1331, 1:500), mouse anti-Ki67 monoclonal (Novo Castra Laboratories, NCL-Ki67-MM1, 1:100), rabbit anti-LIFR polyclonal (Santa Cruz, sc-659, 1:200), mouse anti-Oct3/4 (BD Transduction Laboratories, 611203, 1:300), goat anti-Doublecortin polyclonal (Santa Cruz, sc-8066, 1:200), and rabbit anti-GFAP (Sigma, G9269, 1:400). For immunohistochemistry done on differentiated cells, primary antibodies were mouse anti-O4 monoclonal (Millipore, MAB345, 1:200), rabbit anti-GFAP (Sigma, G9269, 1:400), mouse anti-βIII tubulin monoclonal (Sigma, T8660, 1:400), and mouse anti-human Sox2 (R&D Systems, MAB2018, 1:1000). Secondary antibodies were donkey anti-rat TRITC (Jackson, #712-025-150, 1:200), Alexa Fluor antibodies (Invitrogen) 488 goat anti-rabbit (1:400), 568 donkey anti goat (1:400), 488 goat anti-mouse (1:400), and 568 goat anti-mouse (1:400). Nuclei were counterstained

with DAPI (Vectashield mounting media with DAPI, Vector Labs, H-1200) or Hoechst 33258 (Sigma).

Wholemount sections were derived from *Oct4*-GFP adult mice as previously reported (Mirzadeh et al., 2010). Wholemounts were fixed overnight with 4% PFA in 0.1% TritonX, washed, blocked in 10% NGS with 2% TritonX at room temperature for 1 hour, and primary antibody was chicken anti-GFP (Aves Lab #GFP-1020, 1:500) in the blocking media for 48 hours at 4ºC. Wholemounts were washed and the secondary antibody (Alexa A11039, 1:400) was added in the blocking media for 48 hours at 4ºC. After washing, wholemounts were incubated with Hoechst (Sigma B2261, 1:1000) in St-PBS, washed, and mounted with aquamount and coverslipped. Staining was visualized on an AxioVision Zeiss UV microscope and Nikon 200 microscope or Olympus Fluroview FV1000 confocal laser scanning microscope.

Differentiated EFH neurospheres or LIF colonies were stained for βIII tubulin, GFAP and O4 as described above. Five fields of view were imaged per sample and the number of immunopositive cells and total number of DAPI cells were counted in each view (a minimum of 200 DAPI positive cells for each colony). Relative percent of each cell type per neurosphere/colony was determined by averaging the percentage from each view.

Flow cytometry. *Oct4*-GFP primary cells from the lateral ventricle and cortex, actin-GFP primary cells, and CD1 primary cells were analyzed by flow cytometry. Cells were prepared FACS machine from and following the primary dissection protocol, resuspended in HBSS + 2% FBS with 7AAD to exclude dead cells, and analyzed using a FACS Fortessa (BD).

Single Cell Analysis using Image Stream. Single cells were dissected and prepared as described above. Cells were re-suspended in D-PBS and blocked with 10% NGS (Sigma) in PBS for 1 hour at RT. For live cells, primary rabbit anti-LIFR polyclonal (Santa Cruz, sc-659, 1:200) antibody and Alexa Fluor 568 goat- anti rabbit (1:400) was used for 1 hour at RT consecutively. For fixed cells, 4% PFA was used to fix the cells for 20min at RT. Primary rabbit anti-LIFR polyclonal and mouse anti-GFAP (Sigma, G3893, 1:400) antibody are left for 1 hour at RT followed by Alexa Fluor 568 goat- anti rabbit and 647 goat- anti mouse for 1 hour at RT. Following washing in D-PBS, cells were resuspended in D-PBS before analysis on Amnis Imagestream X. Images are generated using IDEAS software.

Statistics. Data are represented as mean ± sem unless otherwise stated. Statistical analysis were performed by GraphPad Prism 5 (GraphPad Software, Inc., La Jolla, CA) using ANOVA with Bonferroni's multiple comparison test or Student's T-test unless otherwise stated.

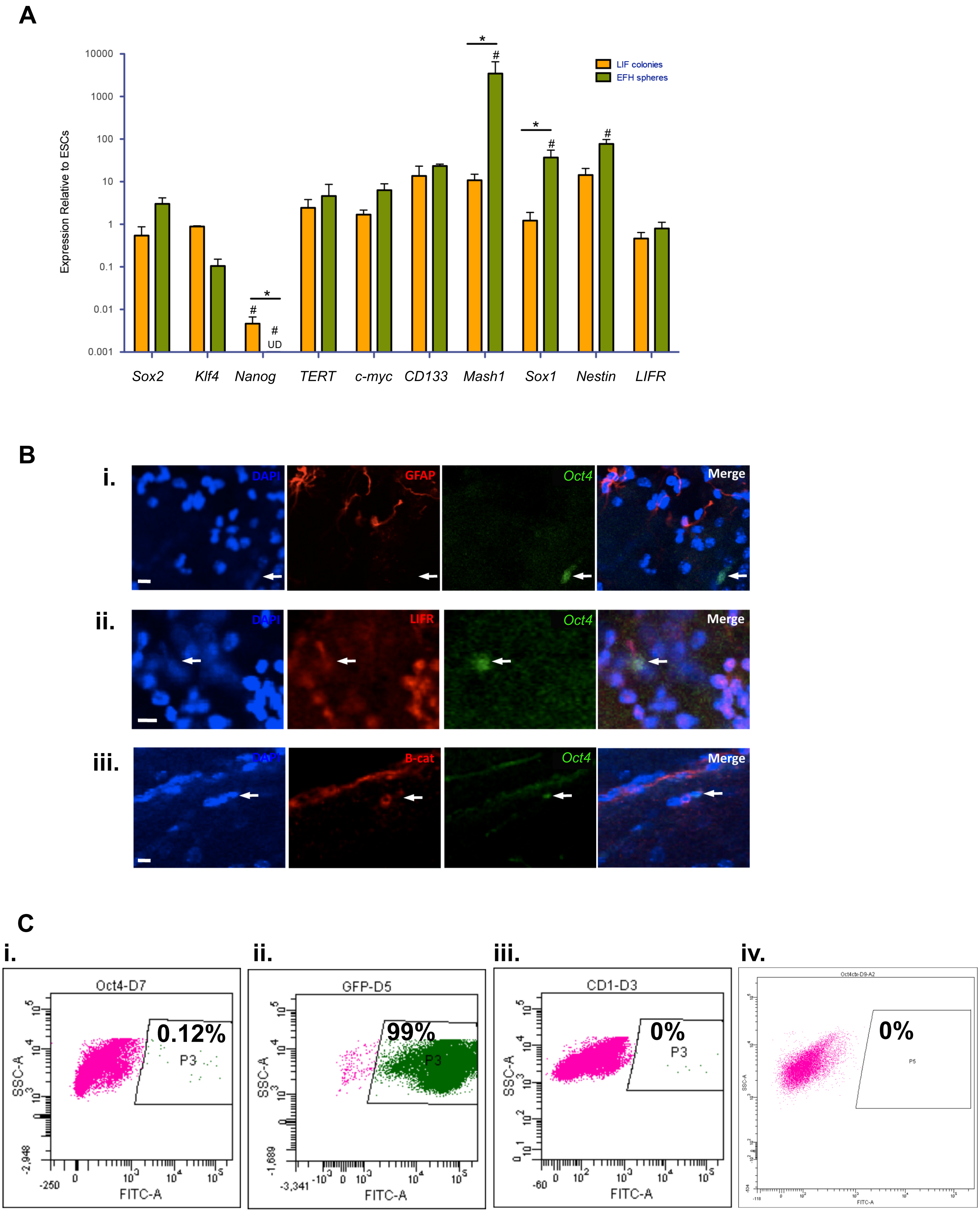

Supplemental Figure 2

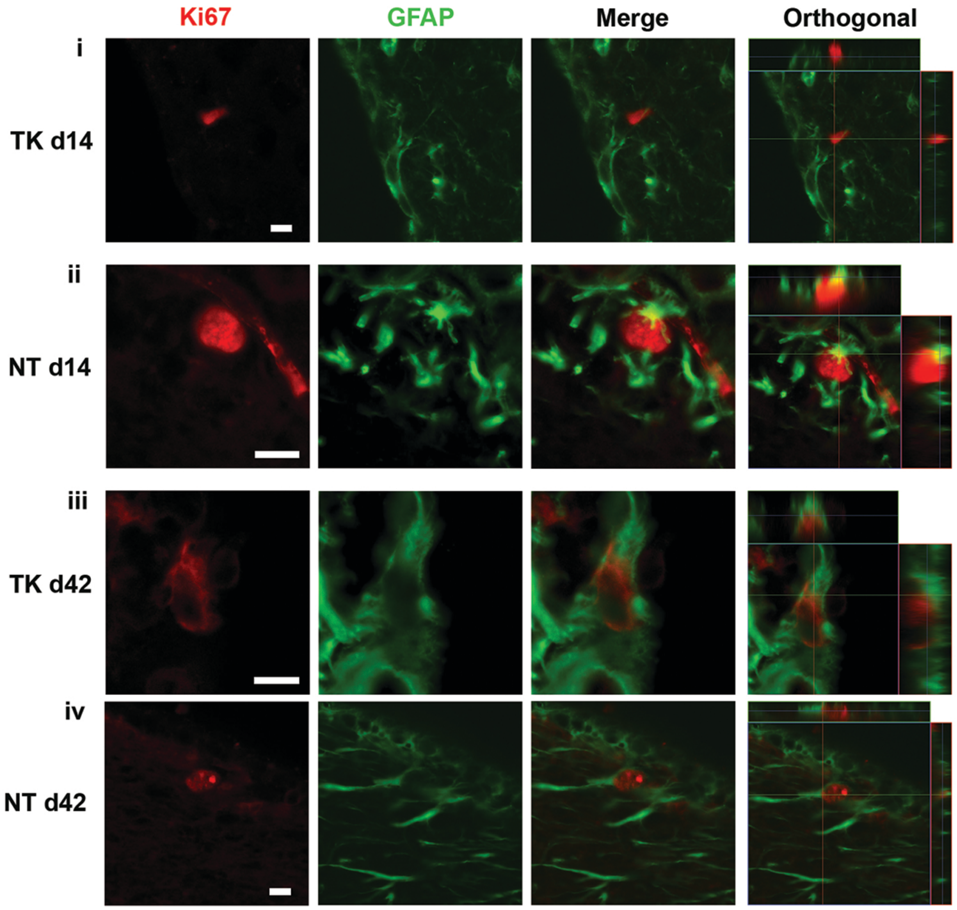

Supplemental Figure 4

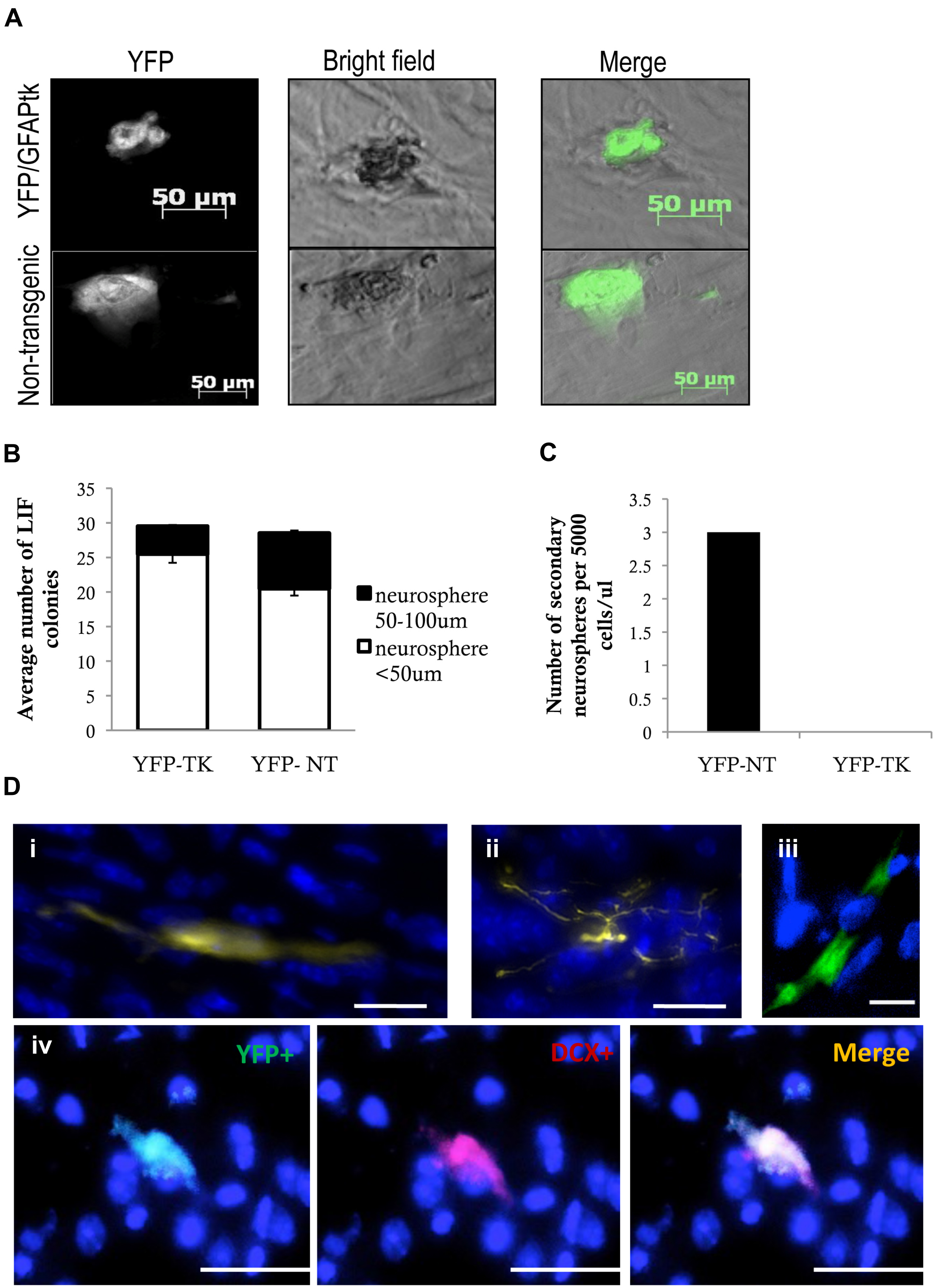

Supplemental Figure 6

SUPPLEMENTAL EXPERIMENTAL PROCEDURES

Animals. GFAP-TK Line 7.1 mice (a kind gift from Dr. M. Sofroniew, UCLA), *Oct4*-GFP mice (a kind gift from Dr. A Nagy, University of Toronto), Floxed *Oct4* mice (a kind gift from Dr. A. Tomlin, UPenn), Sox1-Cre mice (a kind gift from Dr. S. Nishikawa, Japan), YFP reporter mice, adult C57Bl/6 and CD1 (Charles River) mice, and *Oct4*-neo (Jackson Labs) were maintained in the Department of Comparative Medicine at the University of Toronto in accordance with institutional guidelines. GFAP-TK Line 7.1 is well characterized and the transgenic protein alone is not harmful to mammalian cells. Previous analyses at the single cell level on GFAP-TK line 7.1 mice showed 100% of TK expressing cells were also $GFAP^{+}$ in the SE (Garcia et al., 2004; Morshead et al., 2003). GFAP-TK and *Oct4*-neo mice were genotyped employing primer sequences from original source.

Neurosphere Assay. Mice were sacrificed by cervical dislocation. Brains were dissected and the periventricular region was cultured as previously described (Chiasson et al., 1999). For negative control samples, cortical and striatal tissue was sampled from brains and processed at the same time and under identical conditions as periventricular tissue. Cells were plated at clonal density (Coles-Takabe et al., 2008) (10 cells/µL) in 24 well culture plates (Nunclon, 500 µL/well). Standard neurosphere culture conditions consisted of serum free media (SFM) supplemented with epidermal growth factor (EGF, 20 ng/mL; Sigma), basic fibroblast growth factor (bFGF, 10 ng/mL; Sigma) and heparin (2 µg/mL; Sigma). Some cultures were grown in EGF only, LIF only (10.2 ng/mL) (Tropepe et al., 2001). GFAP-TK and controls were plated in the presence or absence of GCV (Sigma, G2536). Cultures derived from *Oct4*-neo mice or controls were plated in the presence or absence of neomycin (G418; 800 µg/ml, Sigma). EFH neurospheres or LIF colonies were counted at 7-10 days *in vitro*.

Self-renewal and differentiation assays. For self-renewal assays, individual neurospheres or colonies were collected and placed in an Eppendorf tube containing 200 *µ*l SFM + appropriate growth factors, triturated, and transferred into 300 µL of media in a 24 well plate to a total volume of 500*µ*l per well. The numbers of neurospheres or colonies was assayed after 7-10 days *in vitro*. For differentiation, individual neurospheres or colonies were plated on Matrigel (1:24 dilution in SFM) in a 24 well plate in SFM with 1-5% fetal bovine serum. After 7-9 days *in vitro*, cells were fixed with 4% PFA for 20 minutes at RT, then washed with PBS.

Stroke. CD1 or YFP transgenic mice were anaesthetized using isofluorane and injected with Ketoprofen (5mg/kg). A rectangular hole was drilled into the frontal and parietal bones running from 0.5 mm posterior to 2.5 anterior to the bregma and running from 0.5 to 3.0 mm lateral from the midline. The dura was removed and a sterile saline soaked cotton swab was used to wipe the blood vessels from the cortical surface. At day 4, 7 and 11 following stroke the mice were killed by cervical dislocation and the brains were dissected and plated for LIF colony formation.

ES and LIF colonies on feeders. Cells were maintained on mouse embryonic fibroblasts (MEFs) in standard ES media supplemented with LIF and passaged once weekly as previously described (Tropepe et al., 2001). ES cell colonies were established from YFP mice and maintained continuously. Adult forebrain cells were derived from YFP or *Oct4*-GFP mice 4 days following stroke and maintained identically to ES cells. After 2-12 passages, the adult derived colonies from YFP mice were used for chimera assay by individual selection of colonies.

GCV and AraC infusions. Mice were anaesthetized with 5% isoflurane, injected with Ketoprofen (5 mg/kg), and a mini osmotic pump (Alzet 1007D, Direct Corp.) was implanted into the right lateral ventricle (+0.2 mm anterior/posterior, +0.7 mm medial/lateral, and 2.5 mm below surface of the skull, relative to bregma). The pump concentration of GCV was 200 μM in sterile PBS. Intraventricular infusion of 2% AraC (Sigma, C1768) in sterile 0.9% saline followed the same protocol.

BrdU labeling. Mice received 3 injections of BrdU (60 mg/kg i.p.; Sigma), 1 every 3 hours, one day prior to sacrifice. $BrdU^+$ cells from each brain region were counted blinded to genotype and from a minimum 3 sections per region per mouse.

Transplantations. Wild-type CD1 adult mice (8-10 weeks) were anesthetized with isoflurane and administered ketoprophen (5mg/kg). Transplant co-ordinates were determined from Bregma at 0.5 lateral, 1.5 anterior, and 2.5 ventral. 1μl of single cell suspension containing 800 YFP-GFAPtk LIF colony-derived cells that had been grown in the presence of GCV for 2 weeks was injected at this location. Animals were allowed to recover and were sacrificed by intracardial perfusions of 4% PFA at 4 hours post-transplantation, 7 days, or 14 days. Brains were post-fixed and cryoprotected in 4% PFA in 20% sucrose overnight and sectioned for YFP cell counts.

qPCR. ES cells and LIF colonies were trypsinized and re-plated. After 30 minutes, the fibroblasts adhered to the plate, and the colonies, which remained suspended, were collected with the media, centrifuged and resuspended in Buffer RLT with β-mercapthenol. EFH neurospheres and pNSC neurospheres were individually picked and collected into Buffer RLT with β-mercapthenol. Fresh tissue was derived from adult mice cortex and periventricular region. Samples were processed as per manufacturer's directions using the RNeasy Micro Kit (Qiagen, Mississauga, ON), including treatment with the RNase-free DNase Set (Qiagen). cDNA synthesis was carried out with Superscript III First Strand Synthesis System (Invitrogen, Carlsbad, CA). QPCR was carried out on a 7900HT Fast Real-Time PCR System (Applied Biosystems). Cycling conditions consisted of initial activation (2 min at 50ºC, then 10 min at 95ºC), followed by 40 cycles of 15s at 95ºC, 1 min at 60ºC, followed by 15s at 95ºC, 15s at 60ºC and 15s at 95ºC. The data were converted into threshold cycles using SDS 2.3 (Applied Biosystems), normalized relative to ES cells and expressed as logarithmic values using Microsoft Excel, and plotted with Graphpad Prism (Graphpad Software, Inc., LaJolla, CA). Taqman Probes: GAPDH (Mm03302249_g1) and *Oct4*/Pou5fl (Mm03053917_g1) Sox2 (Mm03053810_s1), Klf4 (Mm00516104_m1), nanog (Mm02019550_s1), TERT (Mm01352136_m1), c-myc (Mm00451051_g1), CD133 (Mm00477115_m1),

Mash1/Ascl1 (Mm03058063_m1), Sox1 (Mm00486299_s1), Nestin (Mm00450205_m1), LIF-R (Mm00442942_m1) (Applied Biosystems).

Chimera Assay. Morulas were derived from CD1 mice as previously described (Karpowicz et al., 2007) and aggregated with 8-10 cell clusters from various cell types: ES colonies on MEFs, LIF colonies on MEFs, or free-floating EFH neurospheres. The cells and morulas were aggregated overnight at 37ºC in individual aggregation wells. Images were captured during blastoceol cavity formation on a Zeiss Observer D1 with AxioCam MRm.

Astrocyte monolayers. Astrocyte monolayer cultures were generated from neonatal mouse cortex as previously described (Imura et al., 2003). Cells were mechanically dissociated and passed through 40 μm mesh filters and plated in 6 well plates or T25 tissue culture flasks at a density of 50 000 cells/cm^2 in DMEM/F12 (Gibco, Invitrogen) supplemented with 10% FBS. Upon reaching confluence, monolayers were shaken overnight at 200 rpm to remove macrophages and oligodendrocytes, then monolayers were pulsed with final concentration of 20-30 μM GCV for 12 hours. GCV was washed out thoroughly (3 x 10 minute washes) with 1xPBS and media was replaced daily. At 5, 8, 10 and 21 days post-GCV exposure, monolayers were trypsinized and replated in standard neurosphere conditions (EFH).

Tissue preparation and immunohistochemistry. Mice were sacrificed with an overdose of sodium pentobarbital and perfused transcardially with ice cold PBS followed by 4% PFA. Brains were post-fixed overnight at 4ºC then cryoprotected in 20% sucrose until sectioning. Coronal sections (14 μm) were cut on a cryostat (-20 ºC) from the rostral tip of the anterior commissure through the olfactory bulbs. Every 10th section from each brain region (≥5 sections per slide) per region was examined (lateral ventricle, RMS and OB). For immunohistochemistry, sections re-hydrated with PBS and membranes were permeabilized with 0.3% Triton-X in PBS for 20 minutes at RT. For BrdU imaging, DNA was denatured with 1N HCl at 65ºC for 30 minutes. Sections were blocked with 10% NGS (normal goat serum) or 10% NDS (normal donkey serum (Sigma)) in PBS for 1 hour at RT before incubation with primary antibodies at 4ºC overnight. The primary antibodies were rat anti-BrdU monoclonal (Abcam, ab6326 1:100), mouse anti-PSA-NCAM monoclonal (Millipore, MAB5324 1:500), mouse anti-CD133 monoclonal (eBioscience, 14-1331, 1:500), mouse anti-Ki67 monoclonal (Novo Castra Laboratories, NCL-Ki67-MM1, 1:100), rabbit anti-LIFR polyclonal (Santa Cruz, sc-659, 1:200), mouse anti-Oct3/4 (BD Transduction Laboratories, 611203, 1:300), goat anti-Doublecortin polyclonal (Santa Cruz, sc-8066, 1:200), and rabbit anti-GFAP (Sigma, G9269, 1:400). For immunohistochemistry done on differentiated cells, primary antibodies were mouse anti-O4 monoclonal (Millipore, MAB345, 1:200), rabbit anti-GFAP (Sigma, G9269, 1:400), mouse anti-βIII tubulin monoclonal (Sigma, T8660, 1:400), and mouse anti-human Sox2 (R&D Systems, MAB2018, 1:1000). Secondary antibodies were donkey anti-rat TRITC (Jackson, #712-025-150, 1:200), Alexa Fluor antibodies (Invitrogen) 488 goat anti-rabbit (1:400), 568 donkey anti goat (1:400), 488 goat anti-mouse (1:400), and 568 goat anti-mouse (1:400). Nuclei were counterstained

with DAPI (Vectashield mounting media with DAPI, Vector Labs, H-1200) or Hoechst 33258 (Sigma).

Wholemount sections were derived from *Oct4*-GFP adult mice as previously reported (Mirzadeh et al., 2010). Wholemounts were fixed overnight with 4% PFA in 0.1% TritonX, washed, blocked in 10% NGS with 2% TritonX at room temperature for 1 hour, and primary antibody was chicken anti-GFP (Aves Lab #GFP-1020, 1:500) in the blocking media for 48 hours at 4ºC. Wholemounts were washed and the secondary antibody (Alexa A11039, 1:400) was added in the blocking media for 48 hours at 4ºC. After washing, wholemounts were incubated with Hoechst (Sigma B2261, 1:1000) in St-PBS, washed, and mounted with aquamount and coverslipped. Staining was visualized on an AxioVision Zeiss UV microscope and Nikon 200 microscope or Olympus Fluroview FV1000 confocal laser scanning microscope.

Differentiated EFH neurospheres or LIF colonies were stained for βIII tubulin, GFAP and O4 as described above. Five fields of view were imaged per sample and the number of immunopositive cells and total number of DAPI cells were counted in each view (a minimum of 200 DAPI positive cells for each colony). Relative percent of each cell type per neurosphere/colony was determined by averaging the percentage from each view.

Flow cytometry. *Oct4*-GFP primary cells from the lateral ventricle and cortex, actin-GFP primary cells, and CD1 primary cells were analyzed by flow cytometry. Cells were prepared FACS machine from and following the primary dissection protocol, resuspended in HBSS + 2% FBS with 7AAD to exclude dead cells, and analyzed using a FACS Fortessa (BD).

Single Cell Analysis using Image Stream. Single cells were dissected and prepared as described above. Cells were re-suspended in D-PBS and blocked with 10% NGS (Sigma) in PBS for 1 hour at RT. For live cells, primary rabbit anti-LIFR polyclonal (Santa Cruz, sc-659, 1:200) antibody and Alexa Fluor 568 goat- anti rabbit (1:400) was used for 1 hour at RT consecutively. For fixed cells, 4% PFA was used to fix the cells for 20min at RT. Primary rabbit anti-LIFR polyclonal and mouse anti-GFAP (Sigma, G3893, 1:400) antibody are left for 1 hour at RT followed by Alexa Fluor 568 goat- anti rabbit and 647 goat- anti mouse for 1 hour at RT. Following washing in D-PBS, cells were resuspended in D-PBS before analysis on Amnis Imagestream X. Images are generated using IDEAS software.

Statistics. Data are represented as mean ± sem unless otherwise stated. Statistical analysis were performed by GraphPad Prism 5 (GraphPad Software, Inc., La Jolla, CA) using ANOVA with Bonferroni's multiple comparison test or Student's T-test unless otherwise stated.
